# TREM-1 as a potential gatekeeper of neuroinflammatory responses: therapeutic validation and mechanistic insights in experimental traumatic brain injury

**DOI:** 10.3389/fimmu.2025.1636917

**Published:** 2025-07-21

**Authors:** Yunsheng Zhang, Yulian Zhang, Hanhan Dang, Chuanpeng Zhang, Kun He, Xu Yang, Zixi Wang, Li Zhang, Yanbing Yu

**Affiliations:** ^1^ Peking University China-Japan Friendship School of Clinical Medicine, Beijing, China; ^2^ Department of Neurosurgery, China-Japan Friendship Hospital, Beijing, China; ^3^ Department of Neurosurgery, Civil Aviation General Hospital, Beijing, China; ^4^ China-Japan Friendship Hospital (Institute of Clinical Medical Sciences), Chinese Academy of Medical Sciences & Peking Union Medical College, Beijing, China

**Keywords:** traumatic brain injury, TREM-1, neuroinflammation, pyroptosis, NF-κB signaling

## Abstract

**Background:**

Traumatic brain injury (TBI) triggers a cascade of neuroinflammatory responses mediated by microglial activation, which significantly contributes to secondary brain damage. While triggering receptor expressed on myeloid cells-1 (TREM-1) is a key inflammatory amplifier, its mechanistic role and therapeutic potential in TBI remain elusive. Thus, this study endeavored to elucidate the exact role of TREM-1 in experimental TBI.

**Methods:**

A controlled cortical impact (CCI) model was established in male C57BL/6 mice to induce TBI. The temporal expression profile of TREM-1 was assessed using RNA sequencing and Western blot (WB). The TREM-1 inhibitory peptide LP17 was administered intranasally at 2 hours post-injury (hpi). Neurobehavioral assessments, histological analyses, immunofluorescence (IF), brain water content (BWC) measurement, Evans blue (EB) assays and laser speckle contrast imaging (LSCI) were performed in this study. *In vitro* experiments using BV2 microglial cells were conducted to investigate the molecular mechanisms underlying TREM-1-mediated inflammation. Spleen tyrosine kinase (SYK) inhibition was achieved using R406, and TREM-1 small interfering RNA (siRNA) and overexpressing plasmids were performed to validate its role in NF-κB signaling and pyroptosis quantified using WB and enzyme-linked immunosorbent assay (ELISA). Quantitative data are expressed as mean ± SD, and group comparisons were made by two-tailed Student’s t-tests or one-way ANOVA with Tukey’s *post hoc* test, with *P* < 0.05 considered statistically significant.

**Results:**

TBI significantly upregulated TREM-1 expression in microglia, peaking at 3 days post-injury (dpi). Intranasal administration of the TREM-1 antagonist LP17 treatment attenuated neuroinflammation, reduced blood-brain barrier disruption, ameliorated cerebral blood flow decrease, promoted synaptic remodeling, and improved functional outcomes. Mechanistically, through its interaction with SYK, TREM-1 triggered the CARD9/NF-κB signaling and induced pyroptosis. SYK inhibition reversed these effects, confirming the necessity of the TREM-1/SYK axis in neuroinflammatory signaling. *In vitro* studies further demonstrated that TREM-1 overexpression enhanced SYK phosphorylation, NF-κB activation, and pyroptosis, while LP17 or R406 treatment suppressed these responses.

**Conclusion:**

Our finding demonstrates that TREM-1 critically mediates neuroinflammation and synaptic dysfunction after TBI. Pharmacological targeting of TREM-1 attenuates neuroinflammation, reduces cerebral edema, preserves blood-brain barrier integrity, and improves functional recovery. These effects are mediated through suppression of TREM-1-dependent NF-κB signaling and pyroptosis. These results highlight TREM-1 as a promising therapeutic target for TBI.

## Introduction

Traumatic brain injury (TBI) refers to cerebral damage resulting from external mechanical impacts, which may induce neural injury and cause transient or persistent functional deficits (1). TBI is a leading cause of morbidity and mortality worldwide, imposing significant long-term neurological deficits and socioeconomic burdens (2, 3). Hence, identifying efficacious therapeutic interventions for TBI constitutes a crucial research priority.

The pathophysiology of TBI involves complex secondary injury mechanisms, among which neuroinflammatory responses significantly contribute to the aggravation of neuronal injury and subsequent neurological dysfunction (4, 5). As the principal immunocompetent cells within the central nervous system (CNS), microglia are indispensable for maintaining critical neurophysiological processes, including neuronal migration, cellular viability, synaptic remodeling, and myelination mediated by oligodendrocytes (6, 7). Moreover, microglia serve as key mediators of neuroinflammation, contributing to both neuroprotective and neurotoxic effects (8–10). However, the molecular mechanisms regulating microglial activation and their downstream effects on neuroinflammation after TBI remain incompletely understood.

Triggering receptor expressed on myeloid cells-1 (TREM-1) is a well-characterized amplifier of inflammatory responses in peripheral immune cells (11). Emerging evidence suggests that TREM-1 may regulatory role in CNS disorders, potentially modulating microglial activation and neuroinflammation (12–18). However, the specific involvement of TREM-1 in TBI pathogenesis and its therapeutic potential remain largely unexplored. Spleen tyrosine kinase (SYK), a downstream signaling molecule of TREM-1, has been implicated in the regulation of inflammatory pathways, including NF-κB activation and pyroptosis (13). NF-κB serves as a critical regulator of microglial activation and inflammatory responses (19, 20), whereas pyroptosis constitutes an inflammatory-related programmed cell death mechanism (21). However, it remains unclear whether the TREM-1-SYK signaling axis contributes to neuroinflammation and neuronal damage following TBI.

In this study, we aim to elucidate the role and molecular mechanisms of TREM-1 in TBI pathogenesis. Utilizing both *in vivo* controlled cortical impact (CCI) TBI mouse models and *in vitro* lipopolysaccharide (LPS)+ATP stimulated microglia models, we investigate the involvement of TREM-1 in TBI. We further explored the molecular mechanisms by which TREM-1-mediated neuroinflammation, with a particular focus on SYK-dependent signaling pathways, including NF-κB activation and pyroptosis. Our findings provide novel insights into the role of TREM-1 in TBI and highlight its potential as a therapeutic target for alleviating neuroinflammation and improving functional outcomes.

## Materials and methods

### Animals and treatments

Male C57BL/6 mice (7 weeks old, 20–25 g) were obtained from SPF Biotechnology Co., Ltd. (Beijing, China) and acclimatized for one week under controlled conditions (23 ± 2°C, 45–60% relative humidity) with a 12-hour light/dark cycle, given free access to food and water prior to experimental procedures. All experiments were approved by the institutional ethical and safety guidelines (Institutional Animal Welfare and Ethics Committee, China-Japan Friendship Hospital, Beijing, China) (approval ID: ZRDWLL240035) and were implemented according to the National Institute of Health Guide for the Care and the ARRIVE Guidelines 2.0 (Animal Research: Reporting of *In Vivo* Experiments) (22–24). Heat-resistant tools and materials were sterilized by high-pressure steam sterilization (autoclaving at 121°C for 15–20 minutes) and other tools and materials were disinfected using 75% ethanol. At the end of each experiment, mice were humanely euthanized through intraperitoneal administration of sodium pentobarbital (40 mg/kg, 0.3% solution) under deep anesthesia.

### Surgical procedures

Based on previous studies (25–29), a severe TBI was induced using the CCI model. Briefly, mice were anaesthetized with isoflurane (3% induction, 2% maintenance) (RWD Life Science, Shenzhen, China). The mice were subsequently positioned in a prone orientation on a stereotaxic apparatus (RWD Life Science, Shenzhen, China), ensuring the head remained level. Hair was then removed, and the surgical area was disinfected with 75% ethanol. CCI was performed to induce a severe contusion in the left sensorimotor cortex, extending above the hippocampus (impact center: A/P, -2.00 mm; M/L, -2.50 mm from bregma) (27, 30). After performing craniotomy, CCI injury was induced using a standardized TBI induction system (68099II, RWD Life Science, Shenzhen, China) with the following parameters: impact velocity of 3.5 m/s, dwell time of 500 ms, penetration depth of 2 mm, and 3 mm diameter impactor tip. Body temperature was regulated at 37.5 ± 0.5°C throughout the procedure using a feedback-controlled heating system (69001, RWD Life Science, Shenzhen, China).

### Cell culture

The BV2 microglial cell line, obtained from the Cell Bank of the Chinese Academy of Sciences (Shanghai, China), was maintained in Dulbecco’s Modified Eagle Medium (DMEM; Gibco, USA) supplemented with 10% fetal bovine serum (FBS; Gibco, USA) and 1% penicillin-streptomycin solution (Pricella Biotechnology, Wuhan, China). Cell cultures were incubated at 37°C in a humidified atmosphere containing 5% CO2.

### Drug administration

The TREM-1-selective inhibitory peptide LP17 (LQVTDSGLYRCVIYHPP) and its scrambled vehicle peptide (TDSRCVIGLYHPPLQVY) were chemically synthesized by MedChemExpress (MCE, USA). The intranasal administration protocol was established according to previously validated methodologies (12, 14). Specifically, intervention commenced at 2 hours post-injury (hpi) induction through unilateral nostril instillation, with subsequent administrations performed at 24-hour intervals (2, 24, and 48 hpi), constituting a three-dose therapeutic regimen. To optimize dosing parameters, experimental cohorts received LP17 at two distinct concentrations (1.0 μg/g and 3.0 μg/g), while control groups were administered 1.0 μg/g vehicle peptide. SYK inhibition was achieved through intraperitoneal injection of R406 (5 mg/kg, Selleck, USA) administered daily for 3 consecutive days after TBI.

For *in vitro* studies, BV2 cells model were established by exposing cells to LPS (Sigma-Aldrich, USA) and ATP (Sigma-Aldrich, USA). Cells were pretreated with LP17 (1 or 10 μM) for 2 h before LPS + ATP exposure, followed by 24 h LP17 maintenance post-stimulation. SYK inhibition experiments involved pre-incubation with 1 μM R406–2 h prior to LPS + ATP exposure, with continuous R406 treatment maintained throughout the 24 h post-stimulation period. Dose optimization studies systematically evaluated both LPS concentration gradients (0, 1, 2, 5, 10 μg/ml) and LP17 therapeutic windows (1 or 10 μM) at 4mM ATP.

### Cell transfection

For the TREM-1 knockdown experiment, a specific small interfering RNA (siRNA) targeting sequence (5’-GGUAUUCACAGCUGCAUAA-3’) was designed, with a scrambled negative control siRNA serving as experimental control (siNC). For TREM-1 overexpression studies, the pcDNA3.1-TREM-1 overexpression vector (oeTREM-1) and empty vector control (oeNC) were synthesized. All nucleic acid constructs were synthesized by Tsingke Biotechnology (Beijing, China), and Lipofectamine^®^ RNAiMAX regent (Invitrogen, USA) was used as the transfection reagent for siRNA, while Lipofectamine^®^ 3000 reagent (Invitrogen, USA) was utilized for the transfection of overexpression vectors.

### Experimental protocols

The detailed experimental design and methodological workflow are illustrated in **Supplementary Figure 1**.

### Experiment 1

To systematically characterize transcriptional alterations following TBI, twenty-four mice were randomly allocated into eight experimental groups (n = 3 per group): Sham and TBI groups at 2 hpi, 3 hpi, 4 hpi, 6 hpi, 12 hpi, 1 day post-injury (dpi), and 3 dpi. RNA sequencing was performed to profile transcriptome expression changes. Differentially expressed genes (DEGs) were performed using the DESeq2 R package (v4.3.2), with significance determined by adjusted p-values. A stringent threshold (foldchange ≥4; adjusted p-value <0.01) was applied to define significant DEGs. Single-cell RNA sequencing datasets from the GEO database (GSE269748 for 1 dpi; GSE175430 for 3 dpi) were analyzed using a moderated threshold (foldchange ≥2; adjusted p-value <0.05). Overlapping genes across bulk and single-cell datasets were identified through Venn analysis and visualized in a clustered heatmap using the pheatmap R package. Functional annotation of upregulated DEGs was performed using the clusterProfiler R tool to perform Gene Ontology (GO) analyses, with Kyoto Encyclopedia of Genes and Genomes (KEGG) pathway analysis to identify TBI associated biological processes. Protein-protein interaction (PPI) networks were reconstructed via Cytoscape (v3.10.3) to identify hub genes and molecular interplay. The expression levels of key genes were further validated through box plots.

### Experiment 2

To investigate the temporal expression profile and cellular distribution of endogenous TREM-1 at the lesion site following TBI, thirty-five mice were randomly allocated into seven experimental groups (n=5 per group): Sham and TBI-induced groups at 6 hpi, 12 hpi, 1 dpi, 2 dpi, 3 dpi, and 7 dpi. Western blot (WB) analysis was conducted to assess dynamic changes in TREM-1 protein expression levels. Additionally, three independent animals were utilized for immunofluorescence (IF) co-localization studies at the 3 dpi in Sham and TBI groups to determine cell type-specific localization patterns.

### Experiment 3

To determine the optimal therapeutic dosage of the TREM-1-selective inhibitory peptide inhibitor LP17, we conducted a dose-response study in a murine TBI model. Twenty-five mice were randomly allocated into five experimental groups (n=5 per group) at 3 dpi: (1) Sham, (2) TBI, (3) TBI + Vehicle peptide (1 μg/g), (4) TBI + LP17 low-dose (1 μg/g), and (5) TBI + LP17 high-dose (3 μg/g). Following identification of the effective dose, a subsequent cohort of twenty-four mice was utilized for acute neurological assessment. These animals were equally distributed (n=8 per group) into three critical comparison groups: Sham, TBI + Vehicle (1 μg/g), and TBI + LP17 (1 μg/g). Modified neurological severity score (mNSS) (n=8 per group), rotarod test (n=8 per group) and Terminal deoxynucleotidyl transferase-mediated dUTP nick end labeling (TUNEL) staining (n=5 per group) were analyzed.

### Experiment 4

To investigate the long-term neurobehavioral consequences of TREM-1 inhibition, twenty-four mice were randomly assigned to three groups (n = 8 per group): Sham, TBI + Vehicle, and TBI + LP17 (1 μg/g). Behavioral assessments were conducted at standardized time points post-injury, with the open field test (OFT) performed on day 14 to evaluate anxiety-like behaviors, followed by the Morris water maze test from days 15 to 20 to assess spatial learning and memory.

### Experiment 5

To assess the impact of TREM-1 on cerebral edema and blood-brain barrier (BBB) integrity following TBI, seventy-five mice were randomly allocated into three experimental groups (n=25 per group): Sham, TBI+Vehicle, and TBI+LP17 (1 μg/g). Within each group, animals were assigned to specific experimental endpoints: cerebral edema assessment (n=6), Evans Blue (EB) extravasation quantification (n=6), EB fluorescence visualization (n=3), Laser speckle contrast imaging (LSCI) for cerebral blood flow (CBF) analysis (n=5), and WB protein quantification (n=5).

### Experiment 6

To investigate TREM-1 mediated neuroinflammatory responses and microglial polarization dynamics, thirty-three mice were stratified into three cohorts (n=11 per group): Sham, TBI+Vehicle, and TBI+LP17 (1 μg/g). Systematic analysis included IF quantification of microglial polarization markers (CD86+/Iba1+ and CD206+/Iba1+, n=3), polarization and neuroinflammatory markers through reverse transcription quantitative polymerase chain reaction (RT-qPCR) detection of CD16, CD32, iNOS, CXCL-1, CXCL-2, CCL-2, IL-1β and TNF-α in the surrounding brain tissue was injured of TBI (n=3), and IL-1β and IL-18 levels was further quantified via enzyme-linked immunosorbent assay (ELISA) measure (n=5).

### Experiment 7

To investigate the molecular interaction between TREM-1 and SYK, we performed Coimmunoprecipitation (Co-IP) assays using brain tissue lysates from Sham and TBI groups. Spatial co-localization of TREM-1 and SYK was further examined through IF analysis.

To investigate the underlying mechanisms of TREM-1-regulated neuroinflammation after TBI, mice were stratified into three cohorts (n=5 per group): Sham, TBI+Vehicle, and TBI+LP17 (1 μg/g). WB analysis was performed to quantitatively assess the expression levels of key signaling molecules. To establish the essential role of TREM-1 in SYK-mediated pathway activation following TBI, brain tissues from an independent cohort of TBI mice treated with the SYK-specific inhibitor R406 were subjected to WB analysis of the downstream pathways (n=5 per group). Additionally, IL-1β and IL-18 levels were quantified by ELISA to evaluate pyroptosis-associated inflammatory responses (n=5 per group).

### Experiment 8

To address systemic administration limitations *in vivo*, we established a BV2 microglial cells pyroptosis model to investigate TREM-1’s role. We first optimized LPS and LP17 concentrations at 4 mM ATP, then validated *in vivo* findings in BV2 cells. WB analysis assessed Card9-NF-κB pathway and pyroptosis proteins, while ELISA quantified IL-1β and IL-18 release. To further confirm the functional involvement of TREM-1, we developed a siRNA-mediated knockdown system and validated the expression of key proteins in these signaling pathways.

To explore the TREM-1-SYK axis in microglial inflammation, we transfected BV2 cells with oeTREM-1. Four groups were tested: oeNC, LPS + oeNC, LPS + oeTREM-1, and LPS + oeTREM-1 + R406. WB measured the expression of key proteins in NF-κB pathway and pyroptosis, and ELISA quantified IL-1β and IL-18 release.

### Neurobehavior tests

We conducted behavioral tests under low-light conditions. All experimental procedures and data analyses were performed in a blinded manner, with investigators unaware of group assignments.

### Modified neurological severity score

The mNSS was used to assess neurological function according to established protocols (31). This composite evaluation quantifies sensorimotor deficits through three distinct task categories: 1) motor function, 2) reflex response, and 3) balance/coordination. Scoring followed an ordinal scale where one point was assigned per failed task item, ranging from 0 (no neurological deficit) to 14 (maximal impairment). Behavioral assessments were conducted at baseline (24 h pre-operation) and postoperatively on days 1, 2, 3, 5, 7, and 14.

### Rotarod test

The rotarod test was performed to evaluate motor coordination and postural balance following established protocols (27). Briefly, the apparatus was initiated at a baseline rotational velocity of 0 revolutions per minute (rpm), followed by a linear acceleration regimen culminating in 40 rpm in 5 minutes. Test termination occurred upon animal disengagement from the rotating cylinder, with the latency period (defined as time-to-fall) being precisely recorded and quantitatively analyzed.

### Open field test

The OFT was used to assess anxiety-like behavioral as previously reported (32). In brief, each experimental mice was positioned at the central area of the open-field apparatus (500×500×350 mm), and its activity was monitored for a duration of 5 minutes using the LabMaze animal behavior analysis system (Zhongshidichuang Science and Technology, Beijing, China). The total time spent in the center region, along with the total distance traveled throughout the recording period, was meticulously recorded and subjected to quantitative analysis.

### Morris water maze test

The Morris water maze test was used to evaluated spatial learning and memory consolidation over six consecutive days (33). During the navigation phase (15–19 dpi), escape latency was quantified through computerized tracking. Subsequent probe trial analysis on 20 dpi assessed spatial memory retention by measuring target quadrant occupancy duration. Concurrently, motor ability was monitored via mean swim speed (cm/s) calculations. All behavioral parameters were systematically captured and processed through the LabMaze animal behavior analysis system.

### Brain water content

BWC was performed through by wet/dry method as previously described (34). The whole brain was dissected into three distinct regions: ipsilateral hemisphere, contralateral hemisphere, and cerebellum. Each region was promptly weighed using precision microbalances to record wet weight, then dehydrated at 100°C for 24 hours to acquire dry weight measurements. BWC was subsequently calculated using the formula: BWC (%) = ([wet weight−dry weight]/wet weight) ×100.

### Blood brain barrier permeability assays

To measure BBB permeability, 2% EB solution (4 ml/kg) via tail vein injection one hour prior to euthanasia. Mice were intracardiac perfused with 0.9% sodium chloride, followed by brain dissection and weighing. The samples were homogenized in 0.5 ml PBS and 0.5 ml 50% trichloroacetic acid (TCA) solution, then sonicated for 2 minutes. Following overnight incubation of the homogenate at 4°C, the samples underwent centrifugation at 15,000 rpm for 30 minutes at 4°C, and supernatants were collected. Aliquots (100 μl) were analyzed using a spectrophotometer at 620 nm, with quantitative determination achieved through a standardized curve normalized to tissue weight (ng/g). For fluorescence intensity analysis, brain tissues were fixed in 4% paraformaldehyde (PFA) solution at 4°C for 24 hours, followed by preparation of 20 μm coronal sections. EB-derived red autofluorescence was visualized on the slides as previously described (35).

### Laser speckle contrast imaging

Cortical perfusion dynamics following TBI were assessed using LSCI (RWD Life Science, Shenzhen, China). Following deep anesthesia induction, mice were secured in a stereotaxic apparatus with ocular protection provided by erythromycin ophthalmic ointment. A midline scalp incision exposed the skull surface, which was regularly moistened with mineral oil to maintain optical clarity. LSCI recordings were acquired at defined intervals: baseline (pre-injury), 6 hpi, 1 dpi, and 3 dpi. Prior to imaging sessions, a 30-second stabilization period ensured signal consistency. A standardized circular region of interest (ROI, 4 mm diameter) centered on the impact site was established for quantitative analysis. Perfusion metrics were quantified within standardized ROIs and expressed in perfusion units (PU) using manufacturer-calibrated software.

### Histological staining

The mice subjected to intracardiac perfusion with 0.9% sodium chloride followed by 4% PFA after anesthesia. The brains were postfixed in 4% PFA for 24 hours, then dehydrated through a graded sucrose series (10%, 20%, and 30%) at 4°C. Subsequently, the tissues were paraffin-embedded and sectioned at 5 μm sections for subsequent experiments.

### TUNEL staining

The TUNEL staining procedure was performed with the one-step TUNEL apoptosis detection kit (Beyotime Biotechnology, Shanghai, China) according to the manufacturer’s protocol. Briefly, paraffin-embedded tissue sections were deparaffinized in xylene and rehydrated through a graded ethanol series (100%, 95% and 70%). After PBS washing, antigen retrieval was performed using proteinase K (20 mg/mL) at 37°C for 15 minutes. Sections were then incubated with TUNEL reaction mixture (containing TdT and fluorescein-dUTP) at 37°C for 60 min in a light-protected humidified chamber. After termination of the reaction with stop buffer, the sections were stained with 4′,6-diamidino-2-phenylindole (DAPI, 1 μg/mL, Beyotime Biotechnology, Shanghai, China) for 5 minutes to visualize nuclei. Finally, sections were mounted with anti-fade medium and analyzed using fluorescence microscopy.

### Immunofluorescence

Paraffin-embedded tissue sections were initially blocked with a solution containing 0.3% Triton X-100, 10% goat serum, and 1% bovine serum albumin to minimize non-specific binding. Subsequently, sections were incubated with primary antibodies diluted in blocking buffer at 4°C overnight within a humidified chamber. Following three PBS washes, sections were treated with Alexa Fluor 488/594-conjugated secondary antibodies (1:400 dilution, Thermo Fisher Scientific, USA) for 1 hour at room temperature under light-protected conditions. The nuclei were then stained with DAPI for 5 minutes. For each experimental group, tissue analysis was performed using samples from three mice. For each mouse, three sections (5μm thickness) spaced 300 μm apart, spanning the peri-injured cortex were analyzed, with 4–6 non-overlapping fields of view captured per section, and measurements are expressed as the mean value per mouse. ROIs were defined within 1 mm of lesion sites. All fluorescence images were acquired using a Leica STELLARIS 5 microscope (Germany) under consistent exposure settings during quantitative analyses. Cell quantification was performed by an investigator blinded to experimental groups using ImageJ software (v1.5; National Institutes of Health, USA). Measurements are expressed as the mean cells per mm² per mouse ± SD. The specific primary antibodies used in this study are listed in **Supplementary Table 1**.

### RNA isolation and sequencing

Total RNA extraction from tissue samples was performed using TRIzol reagent (Invitrogen, USA) according to the manufacturer’s protocol. RNA quantification was quantified via a NanoDrop 2000 spectrophotometer (Thermo Fisher Scientific, USA). RNA sequencing was provided by OE Biotech Co., Ltd. (Shanghai, China). For cellular RNA isolation, the RNAeasy Animal RNA Isolation Kit with Spin Column (Beyotime Biotechnology, Shanghai, China) was employed following the manufacturer’s guidelines.

### Reverse transcription quantitative polymerase chain reaction

First-strand cDNA synthesis was performed using NovoScript^®^ Plus All-in-one cDNA Synthesis SuperMix (Novoprotein Scientific Inc., China). Next, RT-qPCR was conducted using NovoStart^®^ Fast SYBR qPCR SuperMix (Novoprotein Scientific Inc., Suzhou, China) on the QuantStudio™ 5 Real-Time PCR System (Thermo Fisher Scientific, USA). The experiment was conducted in strict accordance with the manufacturer’s instructions. Primers for the study were independently designed and synthesized by the TsingKe biological technology (Beijing, China), and as listed in **Supplementary Table 2**. mRNA expression levels were quantified relative to GAPDH and the results were calculated using the 2^-ΔΔCt^ method. The data were expressed as fold changes relative to the Sham group *in vivo* or the control group *in vitro*.

### Western blot

Total protein lysates were prepared from brain tissues and cultured cells using radioimmunoprecipitation assay (RIPA) lysis buffer (Beyotime Biotechnology, Shanghai, China) supplemented with protease and phosphatase inhibitors (Beyotime Biotechnology, Shanghai, China), and protein concentration were determined using the BCA protein assay kit (Proteintech, Wuhan, China). Equal amounts of protein were separated by sodium dodecyl sulfate–polyacrylamide gel electrophoresis (SDS-PAGE) (Beyotime Biotechnology, Shanghai, China) and transferred onto polyvinylidene fluoride (PVDF) membranes (Millipore, Boston, MA, USA). After blocking with 5% nonfat milk for 1 h at room temperature, membranes were incubated with primary antibodies at 4°C overnight, and the corresponding HRP secondary antibody was then used for incubation. Immunoblots were visualized using Immobilon Western Chemiluminescent HRP substrate (Abbkine, Wuhan, China) and analyzed with ImageJ software. The specific primary antibodies used in this study are listed in **Supplementary Table 3**, and uncropped original blot images are presented in **Supplementary File 1**.

### Coimmunoprecipitation

The protein immunoprecipitation was performed using a magnetic bead-based immunoprecipitation kit (Beyotime Biotechnology, Shanghai, China) according to the manufacturer’s instructions. Briefly, total cellular lysates were extracted from brain tissues. 500 μg of protein lysates then were incubated with 1 μg of either anti-TREM-1 monoclonal antibody or species-matched control IgG at 4°C for overnight with continuous agitation. After incubation, the mixture was subjected to magnetic separation using a magnetic rack for 10 seconds, after which the supernatant was discarded. Following three cycles of stringent washing with ice-cold TBS containing 0.1% Tween-20, the immunoprecipitated proteins were eluted using SDS-PAGE sample loading buffer and analyzed by WB.

### Enzyme-linked immunosorbent assay

Determination of IL-1β and IL-18 was performed by ELISA as previously described (36). Quantification of inflammatory cytokines was performed using ELISA kits (IL-1β: Solarbio SEKM-0002; IL-18: Solarbio SEKM-0019; Beijing, China). Perilesional brain tissue (2 mm radius from lesion core) was homogenized, followed by centrifugation at 12,000×g for 15 min at 4°C. Cell culture supernatants were collected after 24 hours incubation. All samples were analyzed in technical duplicates according to the manufacturer’s protocol, with absorbance measured at 450 nm (Thermo, Multiskan GO).

### Statistical analysis

Statistical analyses were performed using GraphPad Prism (version 9.5.0) and R software (version 4.3.2). Quantitative data are expressed as mean ± SD. Group comparisons were conducted using two-tailed Student’s t-tests or one-way ANOVA with Tukey’s *post hoc* test. *P* < 0.05 was determined statistically significant.

## Results

### Increased expression of TREM-1 in mouse brain tissue after TBI

**Figure 1:**
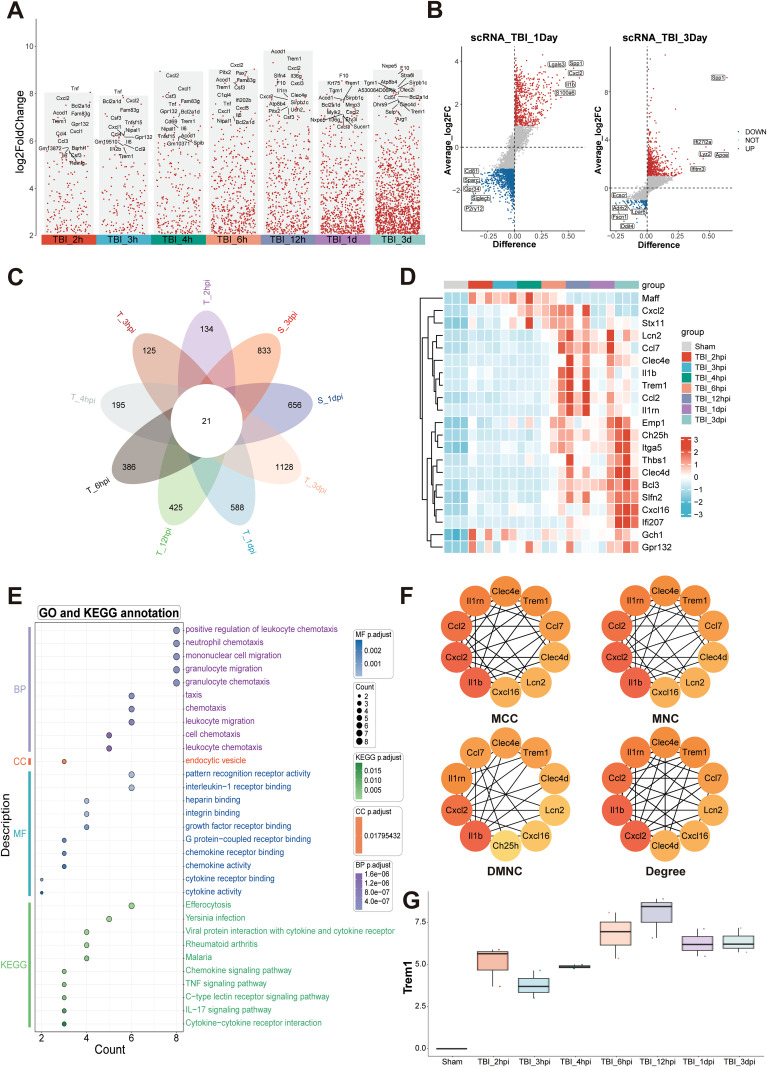
Temporal transcriptomic landscape and inflammatory network activation following TBI. **(A)** Analysis of up-regulated DEGs at multiple time points post-TBI, compared to the baseline with Sham. **(B)** Differential expression analysis of single-cell RNA sequencing data obtained from the GEO database identified genes significantly up-regulated at 1 dpi and 3 dpi. **(C)** Venn diagram analysis of DEGs across multiple TBI time points identified 21 consensus up-regulated genes. **(D)** Heatmap depicting temporal expression patterns of core upregulated genes. **(E)** Functional enrichment analysis highlighting key pathways associated with immune and inflammatory responses. **(F)** Protein-protein interaction network analysis identifying hub genes, including IL1β, Cxcl2, Ccl2, Il1rn, and Trem1. **(G)** Transcriptional analysis of Trem1, showing a significant temporal expression pattern with peak levels at 12hpi.

We conducted a comprehensive comparative analysis of gene expression profiles between the TBI and Sham groups across multiple time points (**Figure 1A**). Comparative analysis against baseline (Sham) identified a cohort of genes exhibiting sustained upregulation, suggesting their potential as key pathological regulators and therapeutic targets in TBI progression. To validate these findings, we integrated single-cell RNA sequencing data from the GEO database. Unsupervised clustering revealed distinct cellular subpopulations (**Supplementary Figures S2A–F**), with subsequent differential analysis confirming temporally upregulated genes at 1 dpi and 3 dpi (**Figure 1B**). Intersectional analysis through Venn diagram identified 21 consensuses DEGs across temporal phases (**Figure 1C**), whose dynamic expression patterns were systematically visualized using a heatmap (**Figure 1D**).

Functional enrichment analysis highlighted the most significant biological processes, including positive regulation of leukocyte chemotaxis, mononuclear cell migration, and granulocyte migration, indicating a robust inflammatory response during the acute phase of TBI (**Figure 1E**). In the cellular components category, “endocytic vesicle” was prominently enriched, reflecting critical cellular structures involved in immune cell functions. Molecular function annotations were enriched in receptor binding-related terms, such as interleukin-1 receptor binding, intergrin binding, chemokine receptor binding and chemokine activity, which are essential for immune signaling. KEGG pathway analysis further identified key signaling pathways associated with immune and inflammatory responses, including chemokine signaling, TNF signaling, C-type lectin receptor signaling, IL-17 signaling pathways, and cytokine-cytokine receptor interactions (**Figure 1E**). Protein-protein interaction network analysis identified several hub genes, including IL1β, Cxcl2, Ccl2, Il1rn, and Trem1 (**Figure 1F**; **Supplementary Figures S2G–J**). These findings collectively underscore the complex temporal dynamics of gene expression and pathway activation following TBI, with Trem1 emerging as a potential key player in these processes. Transcriptional analysis of Trem1 demonstrated a significant temporal expression pattern, with peak levels observed at 12 hpi (**Figure 1G**).

### Temporal expression profile and cellular localization of TREM-1 after TBI

**Figure 2:**
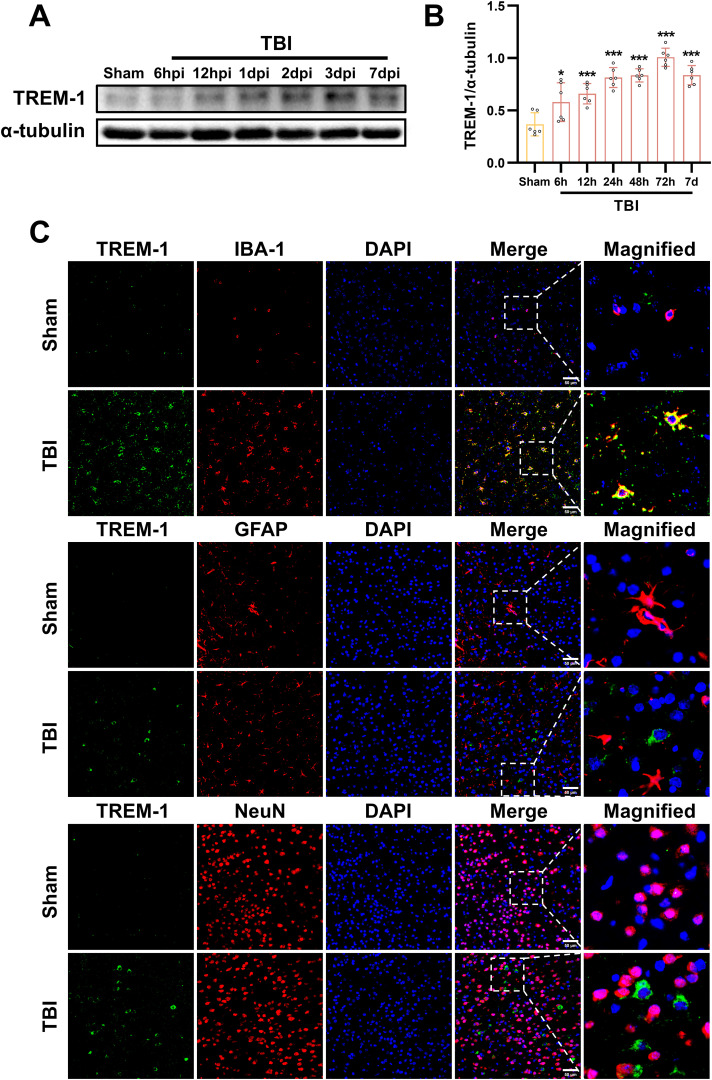
Spatiotemporal profiling of TREM-1 expression in the murine TBI model. **(A)** WB analysis of TREM-1 at the indicated times after TBI, n=5. **(B)** Double immunostaining of TREM-1 with Iba1, GFAP and NeuN at 3 dpi. Nuclei were stained with DAPI (blue), n=3. Scale bar=50 μm.

We next investigated the spatiotemporal protein expression profile of TREM-1 in the murine TBI model. WB results demonstrated a significant upregulation of TREM-1 expression at 6 hpi, peaking at 3 dpi, and followed by attenuation at 7 dpi when compared with Sham group (p < 0.05, **Figure 2A**). Double immunostaining at the peak timepoint (3dpi) demonstrated showed that strong TREM-1 positive cells co-localization with Iba-1, indicating TREM-1 was expressed in microglia. However, no detectable TREM-1 signal was observed in GFAP+ astrocytes and NeuN+ neurons (p < 0.05, **Figure 2B**).

### Inhibition of TREM-1 improved short-term outcomes in TBI mice and reduce neuronal damage

**Figure 3:**
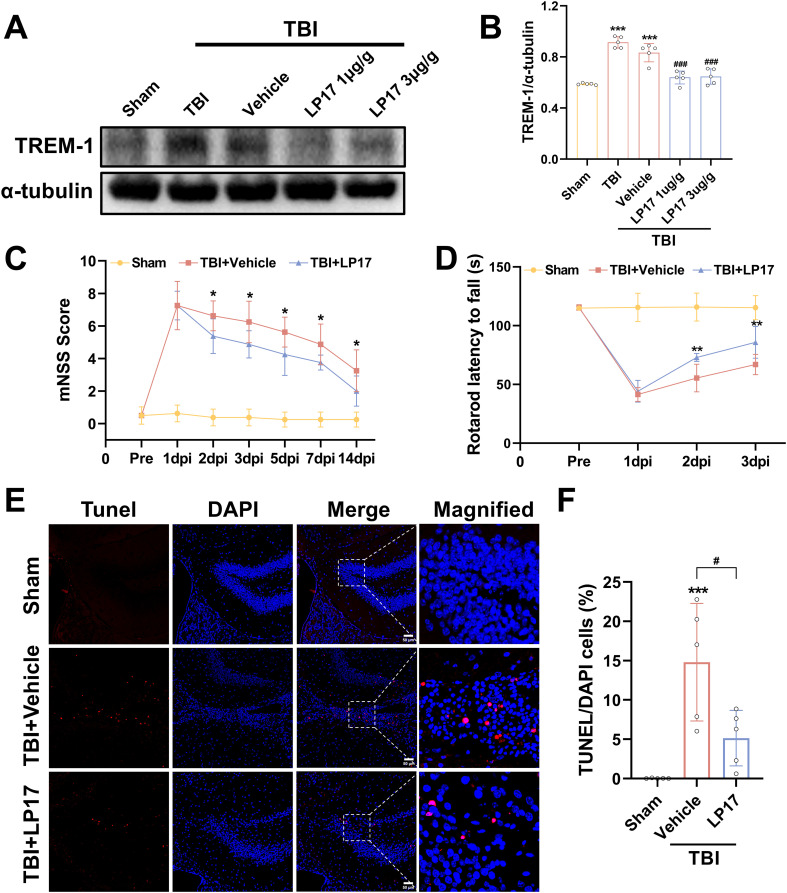
TREM-1 inhibition improves short-term outcomes and reduces neuronal damage in 3 dpi. **(A)** WB analysis of TREM-1 protein expression at 3 dpi following administration of LP17 (1.0 and 3.0 μg/g), n = 5. **(C, D)** Quantitative analysis of neurological function by mNSS **(C)**, rotarod test **(D)**, n=8. *p<0.05, **p<0.01 vs. TBI+Vehicle. **(E, F)** Representative images and quantification of TUNEL-positive cells in the hippocampal DG, n=5. Scale bar=50 μm. ***p<0.001 vs. Sham; # *p*<0.05 vs. TBI+Vehicle.

Dose-dependent pharmacological inhibition of the TREM-1-selective inhibitory peptide LP17 was quantitatively validated at 3 dpi. WB quantification revealed that both LP17 doses (1.0 and 3.0 μg/g) significantly attenuated TREM-1 protein levels compared to the vehicle group (p<0.001, **Figures 3A, B**). Notably, comparative analysis between the two therapeutic concentrations showed no significant dose-response gradient (p>0.05), so subsequent experiments were performed with lower concentrations (**Figures 3A, B**).

Neurobehavioral assessment revealed progressive functional recovery in LP17-treated TBI mice. mNSS analysis demonstrated significant therapeutic divergence beginning at 2 dpi (p<0.05, **Figure 3C**), with sustained improvement through the 14-day monitoring period (p<0.05, **Figure 3C**). Rotarod test corroborated short-term recovery timeline, showing earlier detectable motor coordination benefits in the LP17 cohort. Compared to the vehicle group, LP17 administration significantly prolonged the latency to fall at 2 dpi (p<0.01, **Figure 3D**). This neuroprotective effect persisted at the endpoint assessment (3 dpi, p<0.01, **Figure 3D**).

To further explore the neuroprotective potential of the inhibition of TREM-1, neuronal apoptosis was assessed through TUNEL staining analysis. As shown in **Figures 3E, F**, inhibition of TREM-1 significantly reduced the number of TUNEL positive cells in the dentate gyrus (DG) of the hippocampus compared to the TBI + Vehicle group (p<0.05).

### Inhibition of TREM-1 improved long-term outcomes in TBI mice through synaptic protein modulation

**Figure 4:**
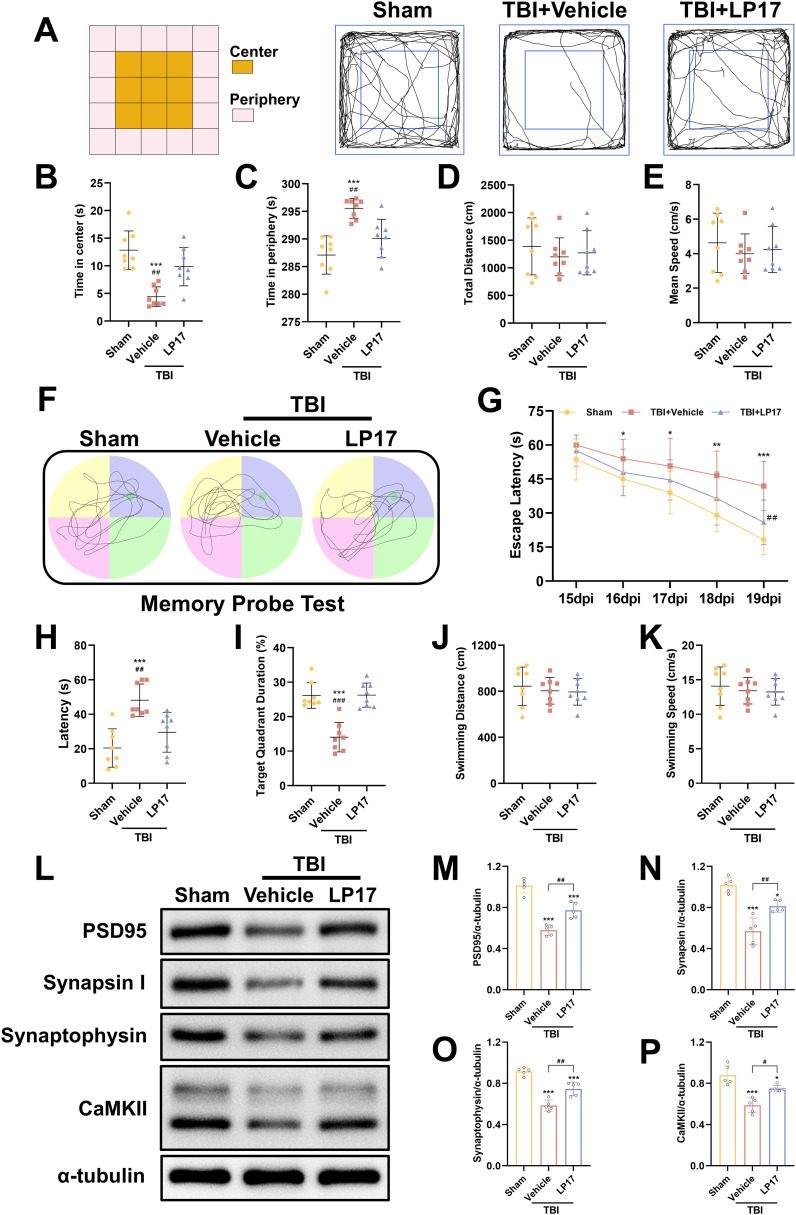
TREM-1 inhibition improves long-term neurobehavioral outcomes and synaptic plasticity in TBI mice. **(A)** Schematic diagram of the open field test and representative movement trajectories of mice at 14 dpi. **(B–E)** Quantitative assessment of behavioral parameters in the open field test, including center time **(B)**, periphery time **(C)**, total distance traveled **(D)**, and mean speed **(E)**, n=8. **(F)** Representative movement patterns from the memory probe test in the Morris water maze. **(G)** Quantitative analysis of escape latency during the learning phase. **(H–K)** Quantification of memory probe test parameters: latency **(H)**, target quadrant duration **(I)**, swimming distance **(J)**, and swimming speed **(K)**, n=8. ***p<0.001 vs. Sham; ##*p*<0.01, ###*p*<0.001 vs. TBI+Vehicle.

To evaluate anxiety-like behaviors at 14 dpi, the open field test was performed. Our results demonstrated that TBI significantly decreased central zone exploration time while increasing peripheral zone activity duration compared to the Sham group (p < 0.05, **Figures 4A–C**), indicating the induction of anxiety-like behaviors. However, inhibition of TREM-1 reversed this effect, TBI + LP17 group significantly increasing the time spent in the center compared to the TBI + Vehicle group (p < 0.01, **Figures 4A–C**). Notably, no significant differences were observed in the total distance or mean speed among the three groups (p > 0.05, **Figures 4D, E**).

To evaluate spatial learning and memory, the Morris water maze test was performed from 15 to 20 dpi. During the learning phase, both the TBI + Vehicle and TBI + LP17 groups exhibited longer latencies to locate the target platform compared to the Sham group (p < 0.05, **Figure 4G**). However, LP17 treatment significantly reduced the latency compared to the TBI + Vehicle group (p < 0.01, **Figure 4G**). In the memory probe test, compared to Sham groups, TBI mice exhibited reduced target quadrant exploration time. However, LP17 administration significantly enhanced target quadrant occupancy relative to the TBI + Vehicle group (p < 0.01, **Figures 4H, I**). No significant differences were observed in the swimming distance or speed during the probe test among the groups (p > 0.05, **Figures 4J, K**).

To further explore the potential mechanisms underlying the functional improvements induced by LP17-mediated TREM-1 inhibition, we evaluated the expression of synaptic proteins at 28 dpi, including PSD95, CaMKII, Synapsin I, and Synaptophysin. WB analysis revealed that TBI significantly suppressed the expression of these synaptic proteins (p < 0.05, **Figures 4L–P**). However, LP17 treatment effectively rescued their expression levels compared to the TBI + Vehicle group (p < 0.05, **Figures 4L–P**). These findings suggest that TREM-1 inhibition ameliorates long-term neurobehavioral deficits through coordinated presynaptic (synaptophysin/synapsin I) and postsynaptic (PSD95/CaMKII) modulation.

### Inhibition of TREM-1 attenuated cerebral edema, BBB disruption, and CBF decrease following TBI

**Figure 5:**
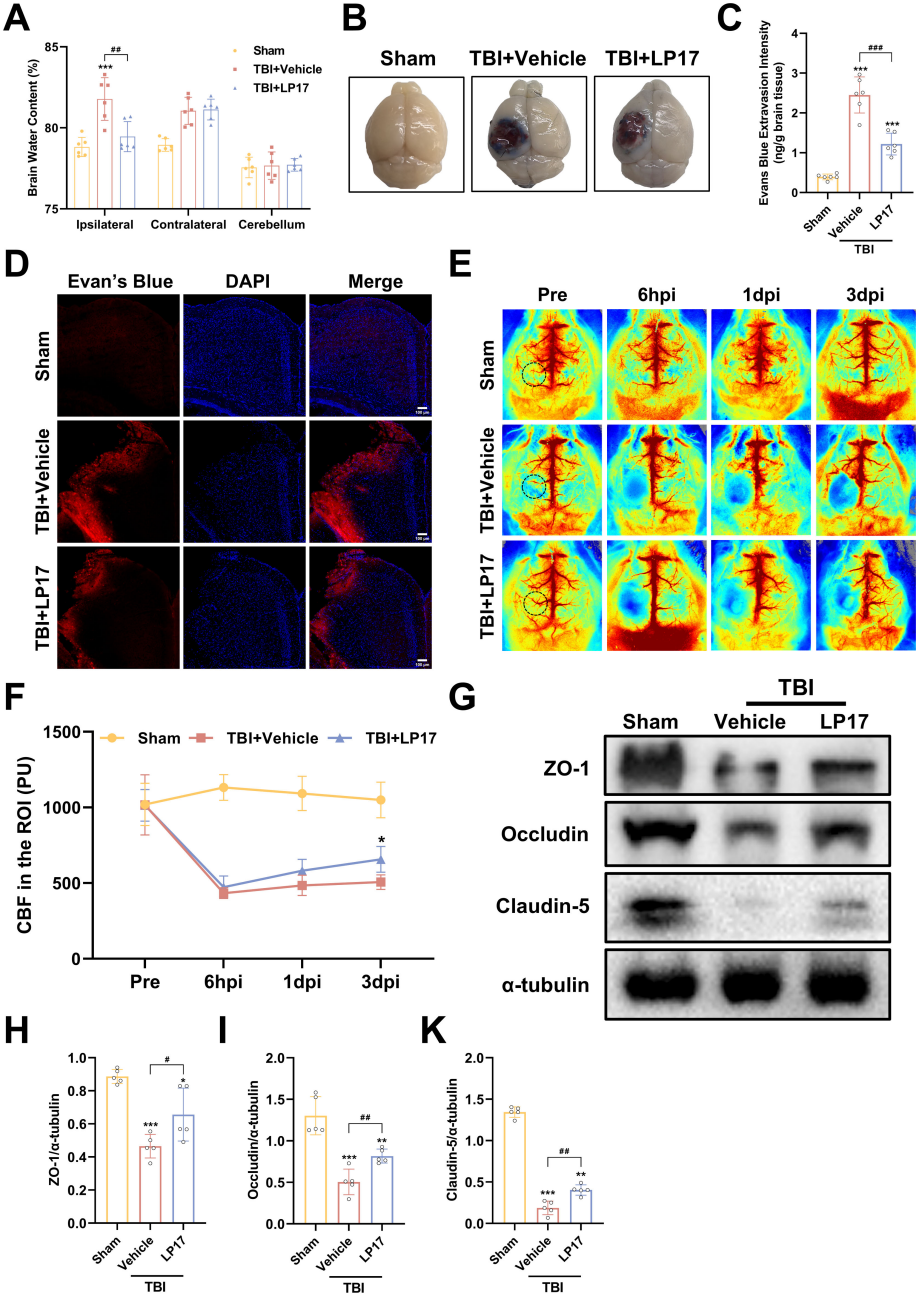
TREM-1 inhibition attenuates cerebral edema, BBB disruption, and improves CBF following TBI. **(A)** Quantification of cerebral edema by wet/dry weight ratio, n=6. **(B)** Representative EB extravasation images. **(C)** Quantitative analysis of EB leakage intensity, n=6. **(D)** Fluorescence microscopy images showing EB extravasation (red) in different groups. Nuclei were stained with DAPI (blue), n=3. Scale bar=100 μm. **(E)** Representative images of CBF assessed by LSCI, black dashed circle indicates ROI. **(F)** Statistical map quantifying CBF changes in peri-lesional cortex, n=5. **(G–K)** WB analysis of tight junction proteins, n=5. *p<0.05, **p<0.01 and ***p<0.001 vs. Sham; *#p<0.05, ##p*<0.01 and *###p*<0.001 vs. TBI+Vehicle.

To assess the therapeutic efficacy of TREM-1 inhibition on cerebral edema, we employed the wet/dry weight method. Our findings revealed significant edema in the ipsilateral hemisphere following TBI, whereas the contralateral hemisphere and cerebellum exhibited no significant changes. Notably, LP17 administration markedly reduced ipsilateral edema compared to the TBI+Vehicle group (p<0.05, **Figure 5A**).

BBB integrity assessment through EB extravasation revealed significantly greater leakage in TBI compared to Sham groups, with LP17 treatment visually reducing EB leakage (**Figure 5B**), as confirmed by quantitative analysis and fluorescence microscopy (p<0.001, **Figures 5C, D**).

LSCI demonstrated significant CBF reduction in the perilesional area following TBI compared to Sham groups, while LP17 administration improved CBF recovery, showing statistically significant improvement at 3 dpi (p<0.05, **Figures 5E, F**).

WB analysis further supported these findings, showing significant downregulation of BBB associated proteins (ZO-1, Occludin, and Claudin-5) in TBI compared to Sham groups, with LP17 treatment promoting better recovery of these proteins relative to the TBI+Vehicle (p<0.05, **Figures 5G–K**). Collectively, these results demonstrate that TREM-1 inhibition through LP17 treatment effectively mitigates TBI-induced cerebral edema and BBB disruption, improves CBF restoration, and promotes recovery of BBB associated proteins.

### Inhibition of TREM-1 attenuates neuroinflammation by modulating microglial polarization and reduce the inflammatory response of microglia

**Figure 6:**
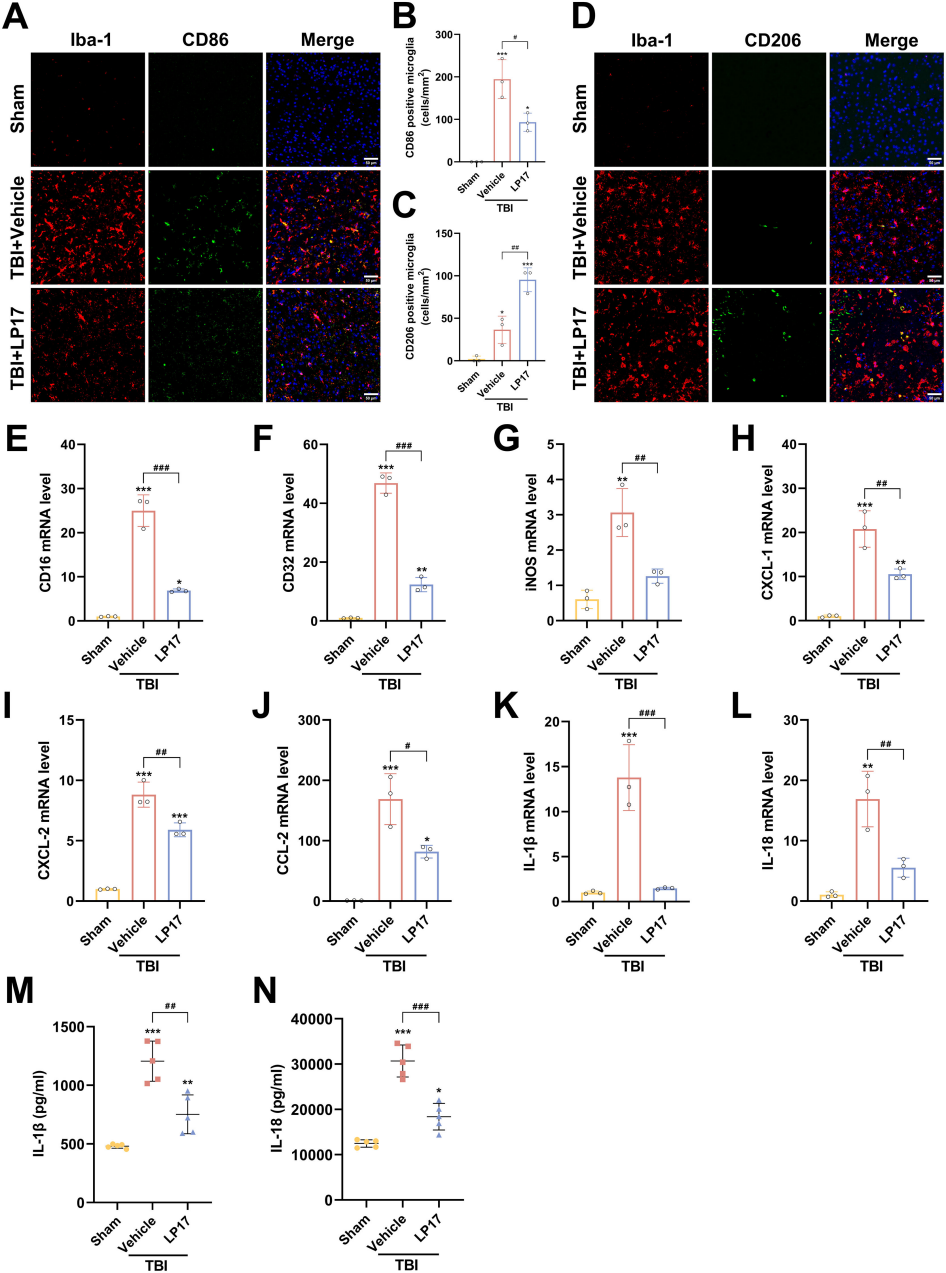
TREM-1 inhibition attenuates neuroinflammation and modulates microglial polarization following TBI. **(A, B)** Representative images and quantification of CD86^+^/Iba1^+^ co-localization (M1 phenotype), n=3. Scale bar=50 μm. **(C, D)** Representative images and quantification of CD206^+^/Iba1^+^ co-localization (M2 phenotype), n=3. Scale bar=50 μm. **(E–L)** RT-qPCR analysis of CD16, CD32, iNOS, CXCL-1, CXCL-2, CCL-2, IL-1β and IL-18 in cortical tissue, n=3. **(M, N)** ELISA quantification of IL-1β and IL-18 levels in cortical tissue, *n* = 5. *p<0.05, **p<0.01 and ***p<0.001 vs. Sham; *#p<0.05, ##p*<0.01 and *###p*<0.001 vs. TBI+Vehicle.

To investigate the role of TREM-1 in post-traumatic neuroinflammation, we assessed the effects of LP17 mediated TREM-1 inhibition on microglial activation and inflammatory responses following TBI. IF analysis revealed a marked elevation in Iba1^+^ microglial density in the TBI group relative to the Sham group at 3 dpi, indicative of robust microglial activation. And LP17 treatment markedly reduced Iba1^+^ cell numbers compared to the TBI+Vehicle group. IF co-localization analysis further revealed that TBI induced microglial polarization toward the pro-inflammatory M1 phenotype, as evidenced by significantly increased CD86^+^/Iba1^+^ co-localization (p < 0.001 vs. Sham, **Figures 6A, B**). In contrast, LP17 administration significantly attenuated CD86^+^/Iba1^+^ co-localization while promoting the expansion of CD206^+^/Iba1^+^ populations (p < 0.01 vs. TBI+Vehicle, **Figures 6A–D**).

Consistent with these findings, RT-qPCR analysis confirmed that TBI upregulated mRNA levels of M1 markers (CD16, CD32, iNOS), which were significantly attenuated by LP17 treatment (p < 0.01, **Figures 6E–G**). Furthermore, RT-qPCR and ELISA revealed a significant upregulation in both mRNA and extracellular protein levels of the proinflammatory cytokines IL-1β and IL-18 following TBI (p < 0.01 vs. Sham, **Figures 6K–N**). LP17 treatment effectively suppressed the transcriptional upregulation and protein secretion of these cytokines (p < 0.01 vs. TBI+Vehicle, **Figures 6K–N**). Similar trends were observed for key chemokines (CXCL-1, CXCL-2, CCL-2), demonstrating significantly elevated mRNA expression levels following TBI, which were subsequently decreased by LP17 treatment (**Figures 6H–J**). Collectively, these data demonstrate that TREM-1 inhibition attenuates neuroinflammation by suppressing microglial activation, modulating M1/M2 polarization, and attenuating the proinflammatory cytokine cascade following TBI.

### TREM-1 interacts with SYK

Previous studies have established that TREM-1 recruits SYK through direct interaction to transduce activation signals to downstream components (15, 36). To determine whether TREM-1-mediated SYK activation occurs following TBI and to investigate the downstream molecular mechanisms, we systematically analyzed DEGs associated with TREM-1 signaling. Comparative analysis between TBI and Sham groups revealed 3,666 significantly differentially expressed genes (**Supplementary Figure S3A**, foldchange>2, adjusted p-value < 0.05). Subsequent comparison between TBI+LP17 and TBI groups identified 327 DEGs (**Supplementary Figure S3B**, foldchange>2, adjusted p-value < 0.05). Using mfuzz clustering analysis, we categorized gene expression patterns into three distinct clusters. Particular attention was given to Cluster 2 (n=13744 genes), which exhibited low expression in Sham group, elevated expression in TBI, and significant downregulation following LP17-mediated TREM-1 inhibition (**Supplementary Figure S3C**). Intersection analysis of these three gene sets yielded 320 core DEGs (**Supplementary Figure S3D**). KEGG pathway enrichment analysis revealed significant associations with immune-regulatory pathways including PI3K-Akt signaling, C-type lectin receptor signaling, TNF signaling, and NF-κB signaling pathways (**Supplementary Figure S3E**). Among these pathways, SYK emerged as a key regulator, showing significant expression changes and pathway alterations. We focused our subsequent analysis on C-type lectin receptor signaling, NF-κB signaling, and NOD-like receptor signaling, based on established roles of SYK in these cascades (12, 13). Gene Set Enrichment Analysis (GSEA) confirmed these pathways were down-regulated after TREM-1 inhibition by LP17 compared with TBI group (**Supplementary Figures S3F–H**).

**Figure 7:**
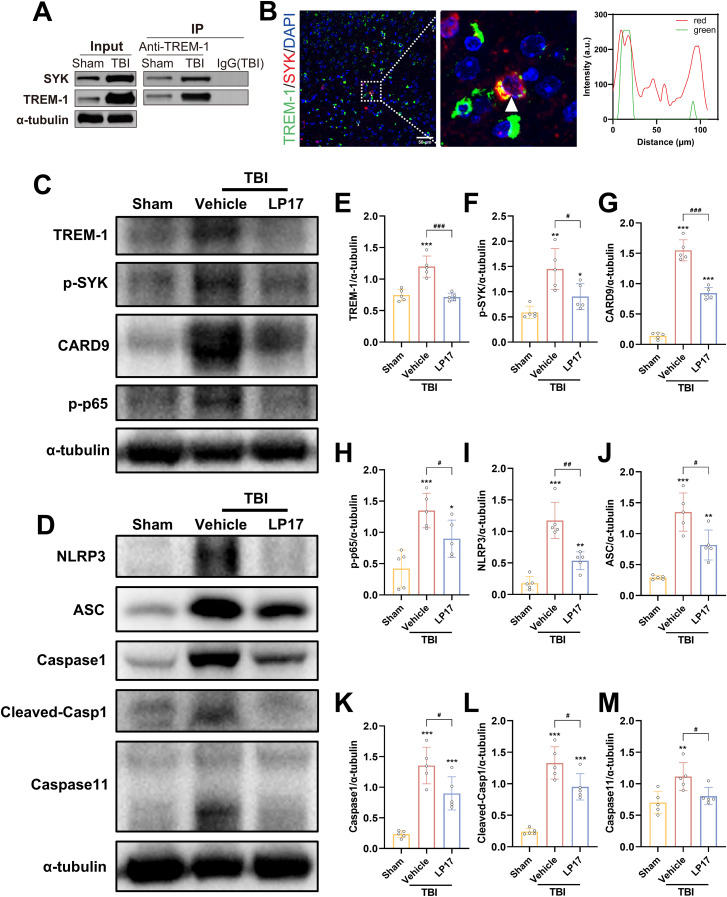
TREM-1 triggered the Card9/NF-κB and pyroptosis pathway through interact with SYK. **(A)** Co-IP of TREM-1 and SYK from Sham and TBI mice. Input lysates and immunoprecipitated complexes were probed with anti-TREM-1 and anti-SYK antibodies. **(B)** IF co-localization of TREM-1 (green) and SYK (red) in peri-lesional cortex at 3 dpi. Nuclei were stained with DAPI (blue). Scale bar=50 μm. Right panel: Quantification for TREM-1/SYK colocalization. **(C–M)** Representative WB images and quantitative analysis of TREM-1, p-SYK, Card9, p-NF-κB p65, NLRP3, ASC, Caspase-1 and Caspase11 in different groups, *n* = 5. *p<0.05, **p<0.01 and ***p<0.001 vs. Sham; *#p<0.05, ##p*<0.01 and *###p*<0.001 vs. TBI+Vehicle.

To validate these findings, we performed Co-IP and IF colocalization assays. Co-IP experiments revealed enhanced TREM-1 and SYK interaction following TBI (**Figure 7A**). IF colocalization studies provided additional evidence of significant TREM-1 and SYK colocalization in TBI affected brain regions (**Figure 7B**).

### TREM-1-induced SYK activates CARD9/NF-κB signaling and pyroptosis in TBI

TREM-1 and phosphorylated SYK (p-SYK) level were significant upregulated in brain tissues following TBI compared to Sham group (**Figures 7C–F**). TBI induced activation of CARD9/NF-κB pathway and canonical pyroptosis, including NLRP3, ASC, and cleaved Caspase-1. Interestingly, Caspase-11 in the non-canonical pyroptosis was also activated after TBI. Notably, pharmacological blockade of TREM-1 with LP17 substantially attenuated these molecular changes, suppressing both SYK phosphorylation and downstream pathway activation (**Figures 7C–M**). These finding suggest that TREM-1 activation triggers SYK activation following TBI, which subsequently amplifies protein expression in both the Card9/NF-κB pathway and the canonical/non-canonical pyroptosis pathways.

**Figure 8:**
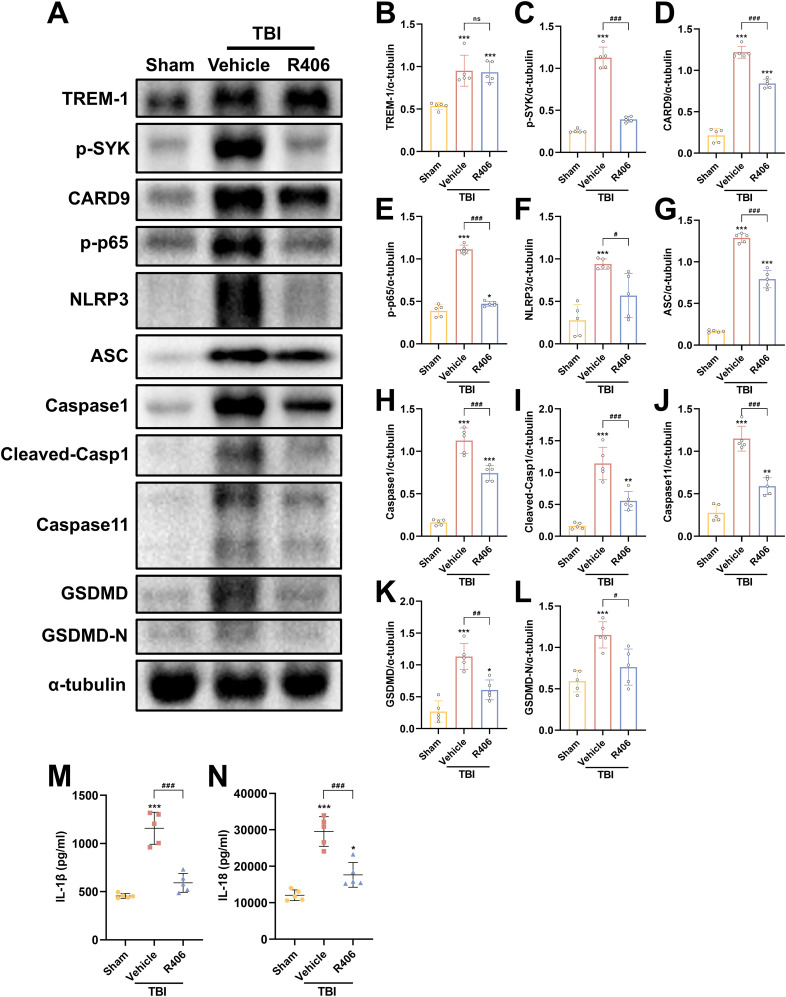
SYK inhibition disrupts TREM-1-dependent signaling cascades. **(A–L)** Representative WB images and quantitative analysis of TREM-1, p-SYK, Card9, p-NF-κB p65, NLRP3, ASC, Caspase-1, Caspase11 and GSDMD in different groups, *n* = 5. **(M, N)** ELISA data demonstrating reduced IL-1β and IL-18 levels in mouse brain tissue following SYK inhibition, *n* = 5. *p<0.05, **p<0.01 and ***p<0.001 vs. Sham; *#p<0.05, ##p*<0.01 and *###p*<0.001 vs. TBI+Vehicle.

To further validate SYK as the pivotal downstream effector molecule in TREM-1 signaling following TBI, SYK-specific inhibitor R406 was administered without affecting TBI-induced TREM-1 elevation. SYK inhibition significantly reversed CARD9/NF-κB activation and pyroptosis protein overexpression (**Figures 8A–L**), including canonical and non-canonical pyroptosis pathways. ELISA data further demonstrated that SYK inhibition can reduce IL-1β and IL-18 levels (**Figures 8M, N**), thereby corroborating the pivotal role of SYK in mediating these inflammatory responses. Taken together, these findings demonstrate that SYK serves as the pivotal downstream effector molecule in TREM-1 signaling following TBI, and further found that TREM-1 can not only regulate changes in the canonical pyroptosis, but also affect the expression of non-canonical pyroptosis.

### TREM-1 activation of NF-κB and pyroptosis in BV2 microglial cells

**Figure 9:**
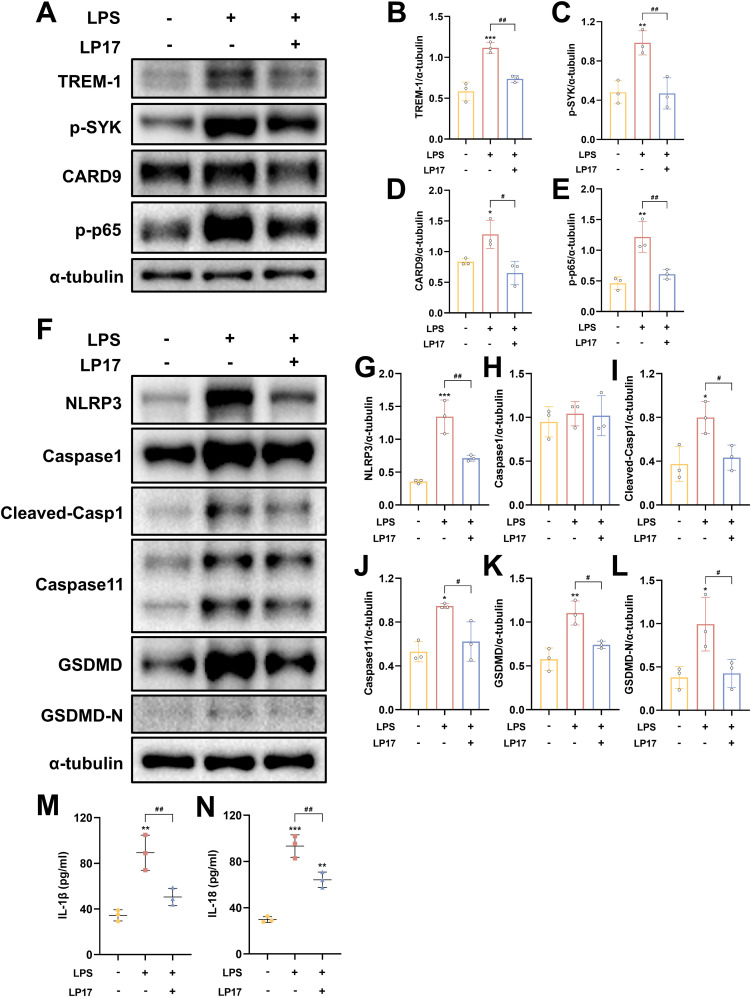
TREM-1 modulates inflammation pathway through SYK-dependent signaling in BV2 microglia cells. **(A–L)** Representative WB images and quantitative analysis of TREM-1, p-SYK, Card9, p-NF-κB p65, NLRP3, Caspase-1, Caspase11 and GSDMD in BV2 microglia cells, *n* = 3. **(M, N)** ELISA data demonstrating reduced IL-1β and IL-18 levels in BV2 microglia cells supernatant following SYK inhibition, *n* = 3. *p<0.05, **p<0.01 and ***p<0.001 vs. Ctrl; *#p<0.05, ##p*<0.01 and *###p*<0.001 vs. LPS+LP17.

Following LPS+ATP treatment, TREM-1 expression in BV2 microglial cells exhibited a dose-dependent increase, peaking at a concentration of 5 μg/ml (**Supplementary Figures S4A, C**). These findings suggest that TREM-1 plays a key regulatory role in mediating microglial inflammatory activation. Additionally, the optimal concentration of LP17 was determined, with 10 μM LP17 significantly inhibiting TREM-1 expression in BV2 cells (**Supplementary Figures S4B, D**). Consistent with the *in vivo* observations, LPS+ATP stimulation significantly upregulated the expression of inflammatory cytokines, which was effectively suppressed by TREM-1 inhibition with LP17 (**Supplementary Figures S4E–L**). WB analysis revealed significant upregulation of key proteins in both the CARD9/NF-κB pathway (CARD9 and p-NF-κB p65) and the pyroptosis pathways (NLRP3, cleaved Caspase-1, Caspase-11, and GSDMD-N) following LPS+ATP stimulation (**Figures 9A–L**). Notably, LP17 treatment effectively attenuated these elevated protein levels, aligning with our *in vivo* results. The release of IL-1β and IL-18 was further confirmed by ELISA (**Figures 9M, N**). To validate these observations, TREM-1 expression was knocked down in BV2 cells using siRNA. WB analysis demonstrated that LPS+ATP-induced upregulation of TREM-1, p-SYK, CARD9, p-p65, NLRP3, cleaved-Casp1 and Caspase 11 were significantly attenuated by TREM-1 siRNA (**Supplementary Figures S5A–I**). The inhibitory effect of TREM-1 knockdown was comparable to that of LP17 treatment. Collectively, these findings indicate that TREM-1 plays a critical role in activating the NF-κB and pyroptosis in BV2 microglial cells.

### TREM-1 drives microglial inflammation through SYK-mediated NF-κB signaling and pyroptosis in BV2 microglial cells

**Figure 10:**
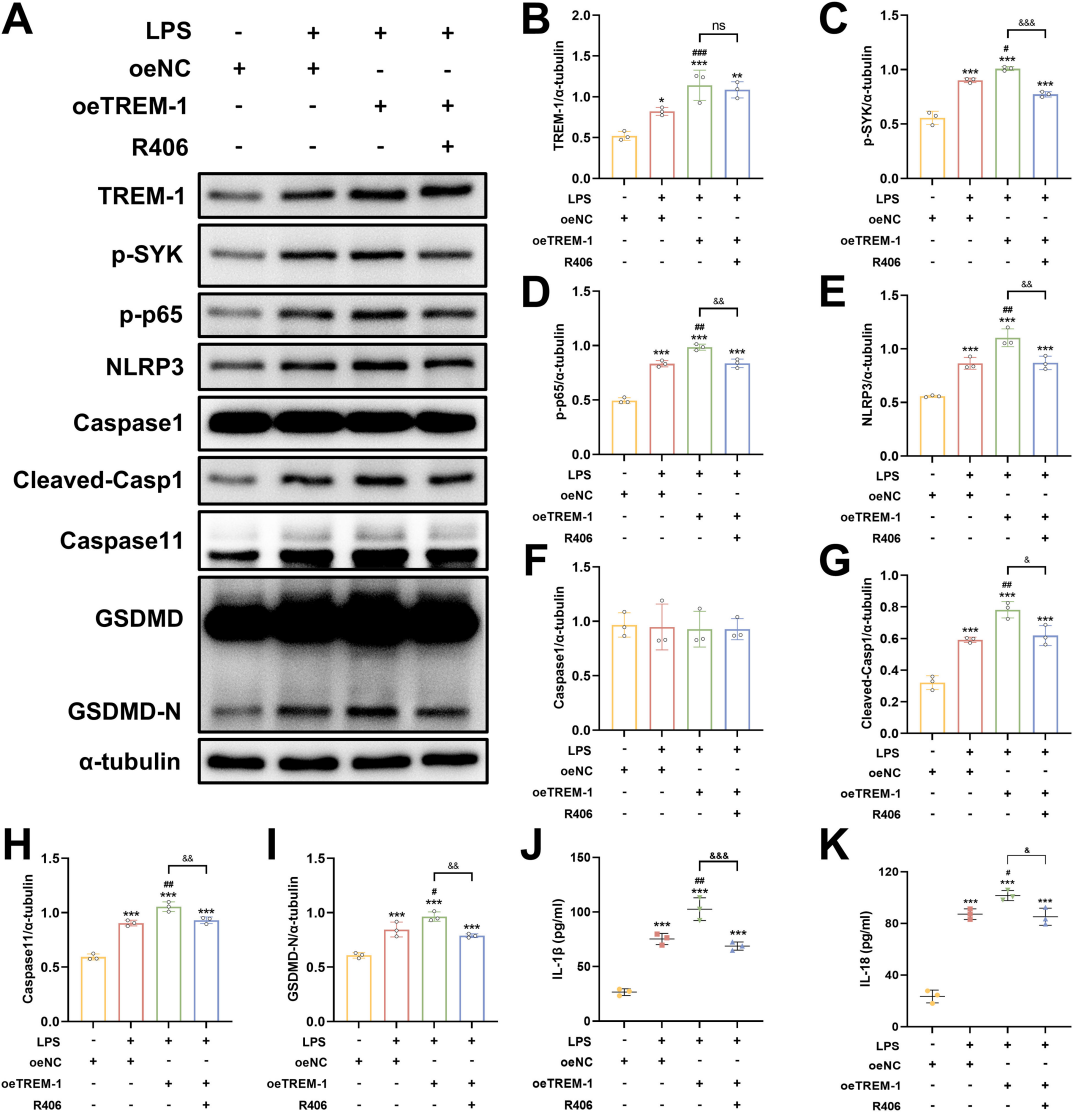
TREM-1-SYK axis regulates microglial inflammatory responses. **(A–I)** Representative WB images and quantitative analysis of TREM-1, p-SYK, p-NF-κB p65, NLRP3, Caspase-1, Caspase11 and GSDMD in BV2 microglia cells, *n* = 3. **(J, K)** ELISA analysis demonstrating that TREM-1 overexpression markedly increased the secretion of pro-inflammatory cytokines IL-1β and IL-18, while R406 treatment significantly suppressed their release in BV2 microglia cells, *n* = 3. *p<0.05, **p<0.01 and ***p<0.001 vs. oeNC; *#p<0.05, ##p*<0.01 and *###p*<0.001 vs. LPS+oeNC; &*p*<0.05, &&*p*<0.01 and &&&*p*<0.001 vs. oeTREM-1; ns, no significance.

To further elucidate the regulatory role of TREM-1-SYK axis in microglial-mediated neuroinflammation, we investigated its impact on NF-κB signaling and pyroptosis in BV2 microglial cells. TREM-1 overexpression in BV2 microglia cells significantly enhanced LPS+ATP-induced SYK phosphorylation and downstream NF-κB activation (p-NF-κB p65), accompanied by the upregulation of pyroptosis factors, including NLRP3, cleaved Caspase-1, Caspase 11 and GSDMD-N (**Figures 10A–I**). While co-treatment with the SYK inhibitor R406 substantially abrogated these effects (**Figures 10A–I**). Consistent with these findings, ELISA analysis demonstrated that TREM-1 overexpression markedly increased the secretion of pro-inflammatory cytokines IL-1β and IL-18, while R406 treatment significantly suppressed their release (**Figures 10J, K**). Collectively, these results indicate that the TREM-1-SYK axis as a critical amplifier of microglial neuroinflammatory responses through coordinated regulation of NF-κB signaling and pyroptosis.

**Figure 11:**
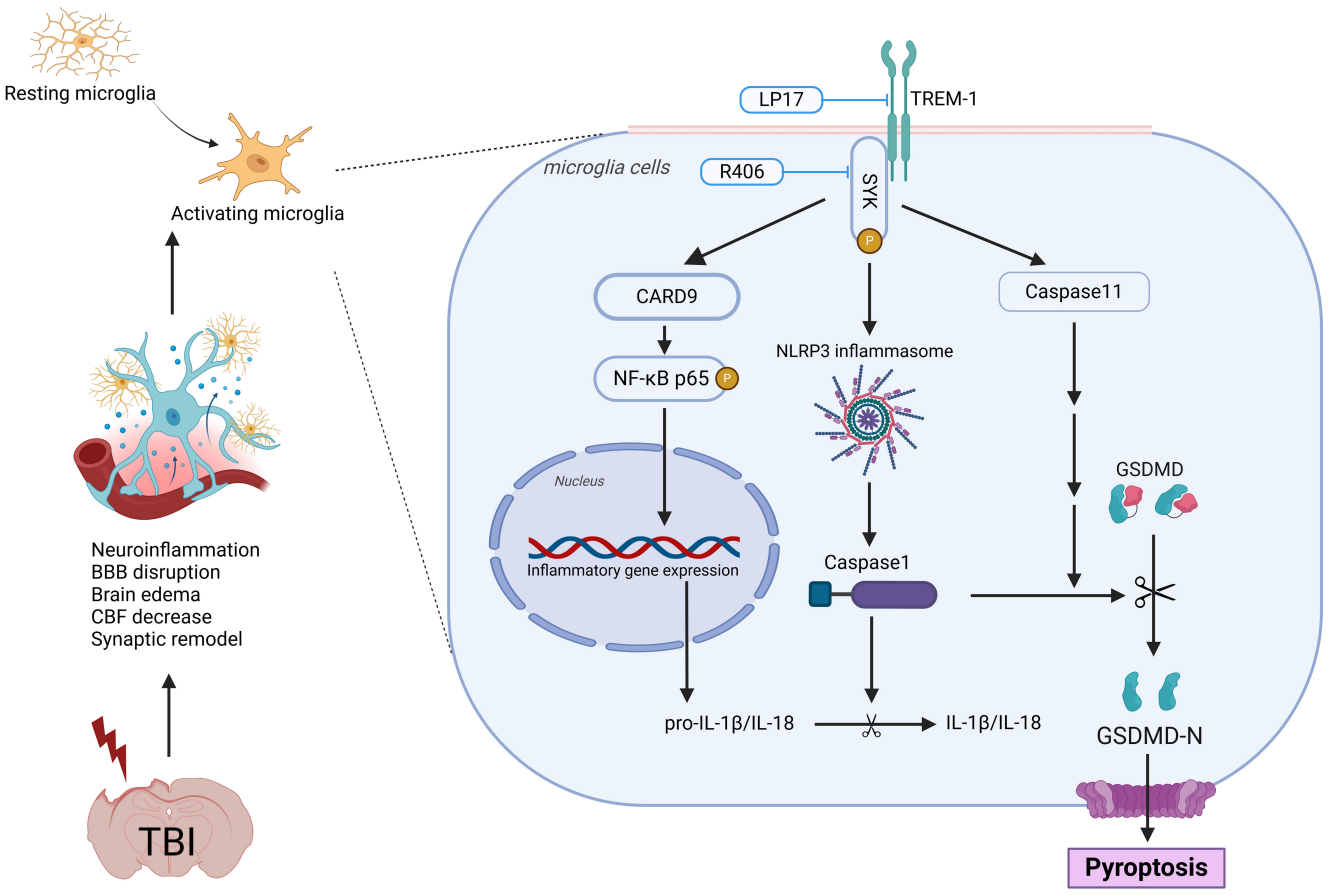
Schematic illustration of the possible mechanisms. The figure was created in https://BioRender.com. Agreement number: JJ27ZGE1ZN.

## Discussion

In this study, we comprehensively investigated the role of TREM-1 in the pathophysiology of TBI and evaluated its potential as a therapeutic target. Our findings revealed a significant upregulation of TREM-1 following TBI, predominantly localized to microglia, the resident immune cells of the CNS. Pharmacological inhibition of TREM-1 using LP17 attenuated neuroinflammation, reduced neuronal apoptosis, attenuated BBB disruption, enhanced CBF recovery, and promoted synaptic remodeling. These findings illuminate the complex interplay between TREM-1 and neuroinflammatory cascades, revealing that TREM-1 blockade not only suppresses microglial activation and the ensuing inflammatory response but also improves both short- and long-term neurological outcomes in murine TBI models. Mechanistically, these effects were mediated through SYK-dependent regulation of CARD9/NF-κB activation and pyroptosis, highlighting the pivotal role of the TREM-1 in driving post-TBI neuroinflammation.

TREM-1 is a transmembrane receptor featuring an extracellular immunoglobulin domain, a transmembrane region, and a short cytoplasmic tail lacking intrinsic signaling motifs (11, 37), was initially identified in peripheral monocytes and neutrophils, with subsequent detection in macrophages, endothelial cells, and fibrosarcoma cell lines (38). TREM-1 exhibits temporally dynamic induction and has been implicated in diverse pathologies, including ischemic stroke, subarachnoid hemorrhage, spinal cord injury, migraine, myocardial infarction, inflammatory bowel disease, and sepsis (11, 13–15, 35, 39–42). While limited studies have explored its role in TBI, recent studies have only found that TREM-1 may affect the polarization state of TBI microglia, but the more in-depth mechanism has not been explored (43, 44). Our integrated transcriptomic and proteomic analyses demonstrated a significant upregulation of TREM-1 expression during the acute phase of TBI, with peak levels observed at 3 dpi. This temporal expression pattern closely consistent with the established kinetics of neuroinflammatory responses following brain injury (13–15, 35, 39, 40). IF further confirmed that TREM-1 activation in the CNS is predominantly confined to Iba-1+ microglia, with negligible expression in GFAP+ astrocytes or NeuN+ neurons. This microglial specificity consistent with prior reports of TREM-1’s role in amplifying inflammatory responses across diverse pathologies (12–16, 35, 39, 40).

TBI triggers a cascade of pathophysiological events leading to persistent neurological deficits. The initial mechanical trauma compromises BBB integrity and disrupts the neurovascular unit, precipitating peripheral immune cell infiltration, microglial activation, astrocytic reactivity, pro-inflammatory cytokine release, oxidative stress, and excitotoxicity-mediated neuronal apoptosis and synaptic dysfunction (8, 9, 45). Neuroinflammation, a cornerstone of these processes, is predominantly driven by activated microglia and astrocytes, and potently contributes to secondary neuronal injury. Emerging evidence positions TREM-1 as a critical modulator of neuroinflammatory responses. Notably, TREM-1 activation exacerbates BBB disruption by promoting NLRP3 inflammasome-mediated endothelial inflammation and apoptosis (13, 17), while upregulating matrix metalloproteinases (e.g., MMP-9) that degrade tight junction proteins (claudin-5, ZO-1) (17, 46). Furthermore, TREM-1 governs microglial polarization toward pro-inflammatory M1 phenotypes, facilitates neutrophil extracellular trap formation (18), and impairs synaptic remodeling—a mechanism potentially linked to β-amyloid phagocytosis exacerbation and cognitive deficits in Alzheimer’s disease (AD) models (43, 47, 48). Elevated TREM-1 levels in AD cerebrospinal fluid further correlate with disease severity (49). However, the mechanisms underlying these processes following TBI and their potential therapeutic implications remain incompletely understood. Furthermore, research on the detailed role of TREM-1 in microglial inflammation is relatively limited, which is the focus of the present study.

The LP17 peptide, a 17-amino acid synthetic decoy receptor mimicking TREM-1’s extracellular complementary-determining region-3 and “F” β-strand (42), effectively bypasses the BBB and allows direct access of therapeutic substances via intranasal administration (12, 14). Our TBI model demonstrated that LP17 treatment suppressed TREM-1, attenuated neuroinflammation, preserved BBB integrity (via restoration of ZO-1, occludin, and claudin-5), improved CBF, and shifted microglial polarization from pro-inflammatory M1 to anti-inflammatory M2 phenotypes. These observations are consistent with previous reports (43). Furthermore, LP17-mediated functional recovery in motor coordination and spatial memory coincided with the preservation of synaptic proteins, reflecting a synergy of its anti-inflammatory properties and modulation of neurotrophic signaling pathways (49). These multifaceted benefits highlight LP17 as a promising therapeutic agent for mitigating TBI-induced damage.

Mechanistically, TREM-1 activation in microglia engages SYK to propagate downstream signaling. Our transcriptomic profiling and experimental validation confirmed SYK co-activation with TREM-1 following TBI. We further found that phosphorylated SYK activates CARD9, which drives NF-κB activation and NLRP3 inflammasome assembly. These pathways have also been demonstrated in previous models involving acute lung injury and cerebral hemorrhage (13, 50–53). Strikingly, we also identified TREM-1-mediated activation of both canonical (NLRP3-caspase-1) and non-canonical (caspase-11) pyroptosis pathways. Pyroptosis, a lytic programmed cell death modality characterized by inflammatory mediator release (21), amplifies neuroinflammation through cytokine/chemokine spillage and immune cell recruitment (54, 55). To our knowledge, this represents the first demonstration of TREM-1’s regulatory role in caspase-11-dependent pyroptosis, unveiling a novel dimension of its pro-inflammatory function in TBI.

While our study provides valuable insights into the role of the TREM-1 in microglial neuroinflammation and its therapeutic potential in TBI, certain limitations should be acknowledged. Firstly, our findings are derived from a murine TBI model, which may not fully capture the complexity of human TBI pathophysiology due to interspecies differences in immune responses, brain anatomy, and injury mechanisms, future investigations should collect clinical data from TBI patients and integrate multi-modal biomarker analyses of tissue, blood, and cerebrospinal fluid samples, enabling comprehensive exploration of TREM-1’s roles in TBI pathogenesis and its diagnostic potential and therapeutic value. Secondly, while our study provides initial evidence for a role of TREM-1 in synaptic remodeling following TBI, this aspect warrants deeper investigation. Future research should leverage conditional TREM-1 knockout mice and integrate multi-omics data (including single-cell and spatial transcriptomics, metabolomics, and proteomics) to delineate the precise mechanisms by which TREM-1 modulates synaptic reorganization and neural circuit repair during recovery.

## Conclusion

Our results demonstrate that TREM-1 plays a critical role in neuroinflammation and synaptic dysfunction following TBI. Inhibition of TREM-1 attenuates neuroinflammation, alleviates cerebral edema and BBB disruption and CBF decrease, promotes synaptic remodeling and improves functional outcomes after TBI. These beneficial effects are achieved by suppressing TREM-1-mediated SYK-dependent activation of NF-κB signaling and pyroptosis. Altogether, our data suggest that TREM-1 represents a potential therapeutic target for TBI.

## Data Availability

The raw data supporting the conclusions of this article will be made available by the authors, without undue reservation.
