# Supplementary material for: TREM-1 as a potential gatekeeper of neuroinflammatory responses: therapeutic validation and mechanistic insights in experimental traumatic brain injury
Source: Front Immunol. 2025 Jul 21;16:1636917. doi: 10.3389/fimmu.2025.1636917 (PMC12318749; doi:10.3389/fimmu.2025.1636917)
Supplement: Supplementary file 1 [file DataSheet1.zip › Supplementary Material/Supplementary Figure Legends.docx]

**Supplementary Figure Legends**

**Supplementary Figure 1:** Experimental design. TREM-1, triggering receptor expressed on myeloid cells 1; TBI, traumatic brain injury; WB, western blot; IF, immunofluorescence; LPS, lipopolysaccharide.

**Supplementary Figure** **2:** Single cell grouping annotation and Cytoscape score table. (A) Dot plot analysis of marker gene expression across distinct cell types in control (CON) and TBI samples at 1 dpi. Dot size represents expression frequency, while color intensity indicates mean expression levels. (B) Bar plot illustrating the proportional distribution of cell types in CON and TBI groups at 1 dpi. (C) UMAP visualization of cell type clustering in CON and TBI at 1 dpi, highlighting altered spatial distribution of macrophages and microglia. (D) Dot plot analysis of marker gene expression across distinct cell types in CON and TBI samples at 3 dpi. Dot size and color intensity represent expression frequency and mean expression levels, respectively. (E) Bar plot depicting the proportional distribution of cell types in CON and TBI groups at 3 dpi. (F) UMAP plots demonstrating spatial reorganization of microglia and macrophages at 3 dpi. (G-J) Tabulated results of network centrality analysis (MCC, MNC, DMNC, Degree), identifying top-ranked genes, including key inflammatory regulators such as IL1β, Cxcl2, Ccl2, Il1rn, and Trem1.

**Supplementary Figure** **3:** Transcriptome analysis reveals TREM-1 inhibition modulates SYK and downstream signaling pathways in TBI. (A-B) Volcano plots of DEGs between (A) TBI vs. Sham and (B) TBI+LP17 vs. TBI groups (foldchange＞2, adjusted p-value < 0.05). (C) Mfuzz clustering of DEGs across experimental groups. Cluster 2 highlights genes upregulated post-TBI and suppressed by LP17 treatment. (D) Venn diagram illustrating overlapping DEGs from three comparisons. (E) KEGG pathway enrichment analysis of core DEGs, identifying significantly enriched pathways associated with TBI pathogenesis. (F-H) GSEA of (F) C-type lectin receptor, (G) NF-κB, and (H) NOD-like receptor signaling pathways between TBI+LP17 and TBI groups.

**Supplementary Figure** **4:** Optimization of LPS and LP17 concentrations and assessment of inflammatory responses in BV2 microglial cells. (A and C) WB and quantitative analysis demonstrating TREM-1 expression in BV2 microglial cells. TREM-1 expression peaked at a concentration of 5 µg/ml LPS in the presence of 4 mM ATP. (B and D) WB and quantitative analysis showing the inhibitory effect of LP17 on TREM-1 expression. LP17 exhibited optimal inhibition at a concentration of 10 µM. (E-L) RT-qPCR analysis of CD16, CD32, iNOS, CXCL-1, CXCL-2, CCL-2, IL-1β and IL-18 in BV2 microglial cells, n=3. **p*<0.05, ***p*<0.01 and ****p*<0.001 vs. Blank control; *#p<0.05, ##p*<0.01 and *###p*<0.001 vs. LPS.

**Supplementary Figure** **5:** TREM-1 knockdown modulates SYK/CARD9/NF-κB signaling and pyroptosis pathways in LPS-treated BV2 microglia cells at 4 mM ATP. (A-I) WB and quantitative analysis were performed to assess protein levels of TREM-1, p-SYK, CARD9, p-NF-κB p65, NLRP3, Caspase 1 and Caspase 11, n = 3. **p*<0.05, ***p*<0.01 and ****p*<0.001 vs. Blank control; *#p<0.05, ##p*<0.01 and *###p*<0.001 vs. LPS.
