# Supplementary material for: TREM-1 as a potential gatekeeper of neuroinflammatory responses: therapeutic validation and mechanistic insights in experimental traumatic brain injury
Source: Front Immunol. 2025 Jul 21;16:1636917. doi: 10.3389/fimmu.2025.1636917 (PMC12318749; doi:10.3389/fimmu.2025.1636917)
Supplement: Supplementary file 1 [file DataSheet1.zip › Supplementary Material/Supplementary Table 1.docx]

**Supplementary Table 1**: Antibodies used in immunofluorescence staining

| **Antibody** | **Host** | **Source** | **Dilutions** |
| --- | --- | --- | --- |
| TREM-1 | Rabbit | Proteintech (11791-1-AP) | 1:100 |
| Iba-1 | Rabbit | Wako (PAP2503) | 1:300 |
| GFAP | Rabbit | Abcam (Ab68424) | 1:500 |
| NeuN | Rabbit | Abcam (177487) | 1:300 |
| CD86 | Rabbit | CST (19589S) | 1:400 |
| CD206 | Rabbit | Abcam (Ab64693) | 1:1000 |
| SYK | Rabbit | CST (13198) | 1:200 |
