# Supplementary material for: TREM-1 as a potential gatekeeper of neuroinflammatory responses: therapeutic validation and mechanistic insights in experimental traumatic brain injury
Source: Front Immunol. 2025 Jul 21;16:1636917. doi: 10.3389/fimmu.2025.1636917 (PMC12318749; doi:10.3389/fimmu.2025.1636917)
Supplement: Supplementary file 1 [file DataSheet1.zip › Supplementary Material/Supplementary Table 2.docx]

**Supplementary Table 2**: Primers used in real-time PCR

| Gene | Sense Primer (5'-3') | Antisense Primer (3'-5') |
| --- | --- | --- |
| CD16 | TTTGGACACCCAGATGTTTCAG | GTCTTCCTTGAGCACCTGGATC |
| CD32 | AATCCTGCCGTTCCTACTGATC | GTGTCACCGTGTCTTCCTTGAG |
| iNOS | GTTCTCAGCCCAACAATACAAGA | GTGGACGGGTCGATGTCAC |
| CXCL-1 | GCCACACTCAAGAATGGTCG | CTTGGGGACACCTTTTAGCA |
| CXCL-2 | CCAGACAGAAGTCATAGCCACT | ATGATTTTCTGAACCAGGGGG |
| CCL-2 | CACTCACCTGCTGCTACTCA | GCTTGGTGACAAAAACTACAGC |
| IL-1β | TGTAATGAAAGACGGCACACC | TCTTCTTTGGGTATTGCTTGG |
| IL-18 | ACCACTTTGGCAGACTTCACT | ACACAGGCGGGTTTCTTTTG |
| GAPDH | AAGAAGGTGGTGAAGCAGG | GAAGGTGGAAGAGTGGGAGT |
