# Supplementary material for: TREM-1 as a potential gatekeeper of neuroinflammatory responses: therapeutic validation and mechanistic insights in experimental traumatic brain injury
Source: Front Immunol. 2025 Jul 21;16:1636917. doi: 10.3389/fimmu.2025.1636917 (PMC12318749; doi:10.3389/fimmu.2025.1636917)
Supplement: Supplementary file 1 [file DataSheet1.zip › Supplementary Material/Supplementary Table 3.docx]

**Supplementary Table 3:** Antibodies used in western blot

| **Antibody** | **Host** | **Application** | **Source** | **Dilutions** |
| --- | --- | --- | --- | --- |
| TREM-1 | Rat | WB | R&D (MAB1187) | 1:500 |
| SYK | Rabbit | WB | CST (13198) | 1:1000 |
| p-SYK | Rabbit | WB | CST (2710) | 1:1000 |
| CARD9 | Rabbit | WB | CST (77568) | 1:1000 |
| p-NF-κB p65 | Rabbit | WB | CST (3033) | 1:1000 |
| NF-κB p65 | Rabbit | WB | Selleck (F0006) | 1:1000 |
| NLRP3 | Rabbit | WB | CST (15101) | 1:1000 |
| ASC | Rabbit | WB | Selleck (F0468) | 1:1000 |
| Caspase-1 | Mouse | WB | AdipoGen (AG-20B-0042) | 1:1000 |
| Caspase-4 | Rabbit | WB | Abcam (ab180673) | 1:1000 |
| Gasdermin D | Rabbit | WB | Abcam (ab209845) | 1:1000 |
| Occludin | Rabbit | WB | Selleck (A5381) | 1:1000 |
| Claudin-5 | Rabbit | WB | Selleck (F1668) | 1:1000 |
| ZO-1 | Rabbit | WB | Proteintech (21773-1-  AP) | 1:1000 |
| PSD95 | Rabbit | WB | Selleck (F0240) | 1:1000 |
| CaMKII | Rabbit | WB | Selleck (F1032) | 1:1000 |
| Synapsin I | Rabbit | WB | Selleck (F0455) | 1:1000 |
| Synaptophysin | Rabbit | WB | Selleck (F1215) | 1:1000 |
| α-tubulin | Rabbit | WB | Proteintech (HRP-66031) | 1:10000 |
| Anti-mouse IgG (H+L) | Goat | WB | Proteintech (SA00001-1) | 1:5000 |
| Anti-rabbit IgG (H+L) | Goat | WB | Proteintech (SA00001-2) | 1:5000 |
| Anti-rat IgG (H+L) | Goat | WB | Solarbio (5124207001) | 1:5000 |
| Anti-Rabbit IgG, Light Chain Specific | Mouse | IP | Proteintech (SA00001-7L) | 1:5000 |
