# Supplementary material for: TREM-1 as a potential gatekeeper of neuroinflammatory responses: therapeutic validation and mechanistic insights in experimental traumatic brain injury
Source: Front Immunol. 2025 Jul 21;16:1636917. doi: 10.3389/fimmu.2025.1636917 (PMC12318749; doi:10.3389/fimmu.2025.1636917)
Supplement: Supplementary file 1 [file DataSheet1.zip › Supplementary Material/Supplementary file 1.pdf]

**Supplementary file for**

# TREM-1 as a potential gatekeeper of neuroinflammatory responses: therapeutic validation and mechanistic insights in experimental traumatic brain injury

Yunsheng Zhang[1,2,3], Yulian Zhang[2], Hanhan Dang[2,4], Chuanpeng Zhang[1,2], Kun He[1,2], Xu Yang[1,2], Zixi Wang[1,2], Li Zhang[1,2,4], Yanbing Yu[1,2,4*]

**This file includes Original Blots:**

Original Blots areas for blots in the main figures are shown with a line box.

Figure 2A

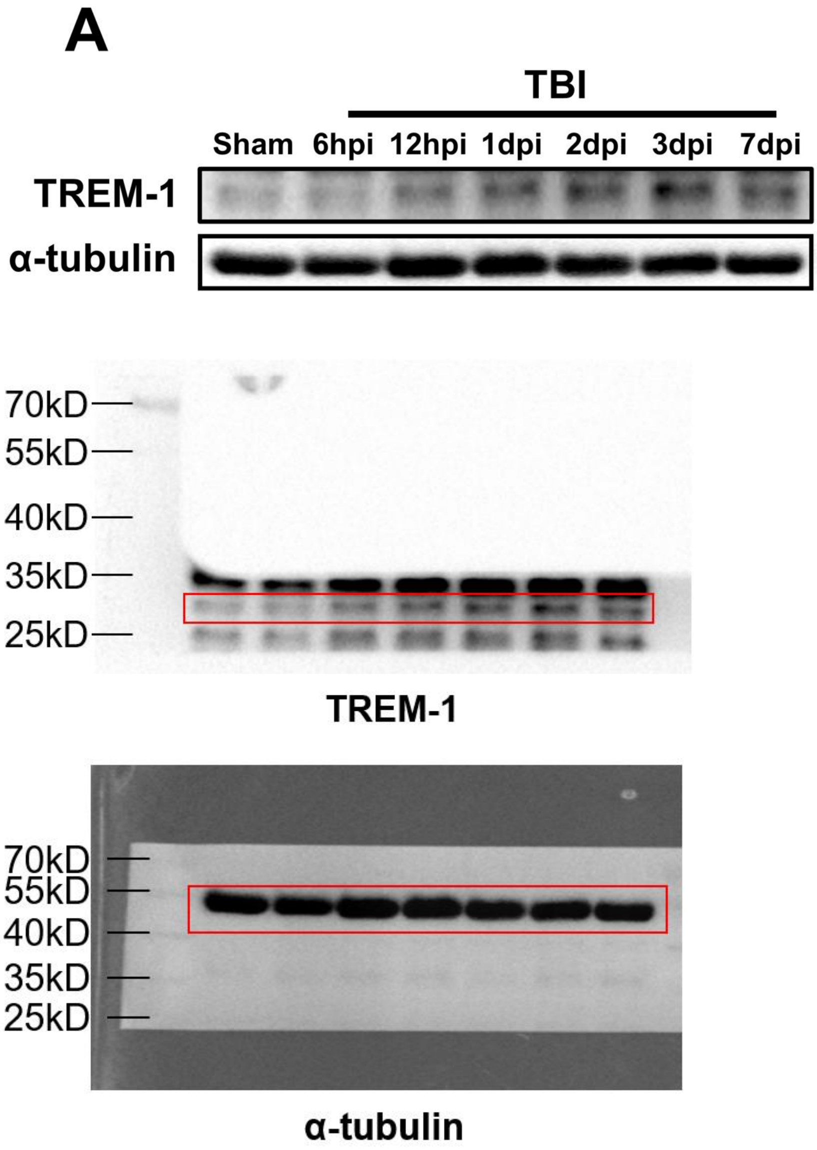
A
TBI
Sham
6hpi
12hpi
1dpi
2dpi
3dpi
7dpi
TREM-1
α-tubulin
70kD
55kD
40kD
35kD
25kD
TREM-1
70kD
55kD
40kD
35kD
25kD
α-tubulin

Figure 3A

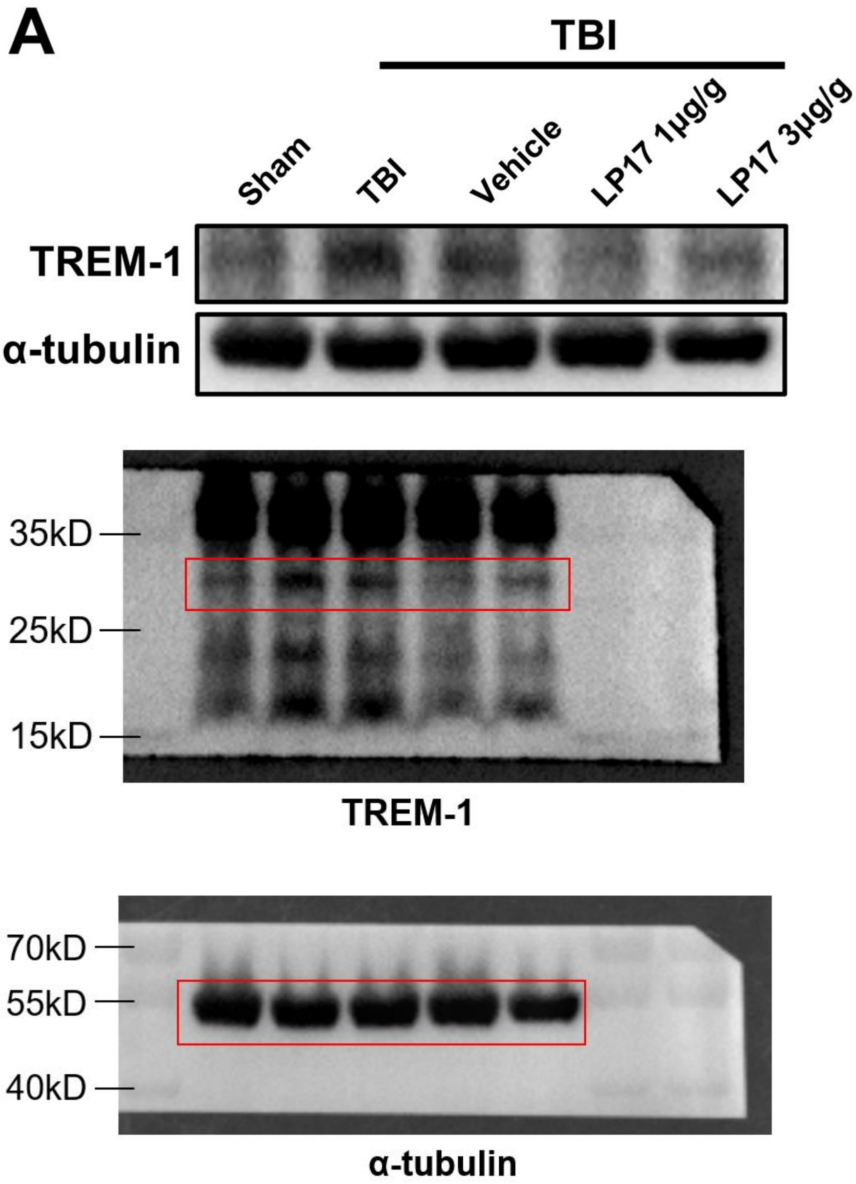

A
TBI
Sham
TBI
Vehicle
LP17 1μg/g
LP17 3μg/g
TREM-1
α-tubulin
35kD
25kD
15kD
TREM-1
70kD
55kD
40kD
α-tubulin

**Figure 4L**

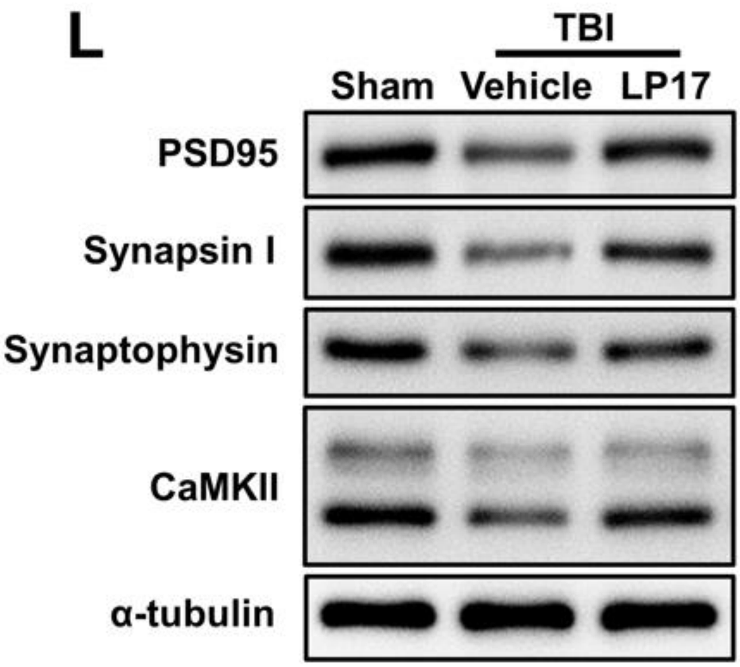

L
TBI
Sham
Vehicle
LP17
PSD95
Synapsin I
Synaptophysin
CaMKII
α-tubulin


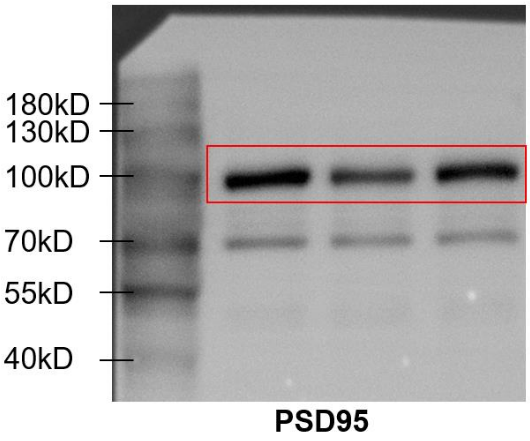

180kD
130kD
100kD
70kD
55kD
40kD
PSD95


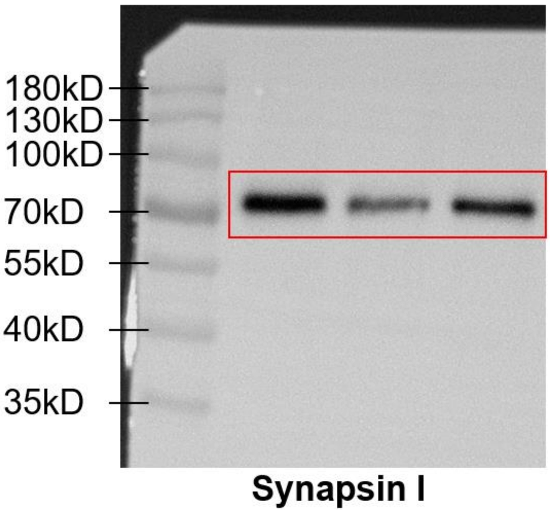

180kD
130kD
100kD
70kD
55kD
40kD
35kD
Synapsin I

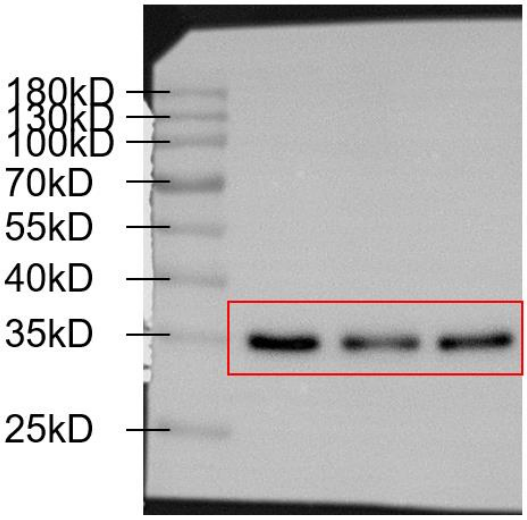


**Synaptophysin**

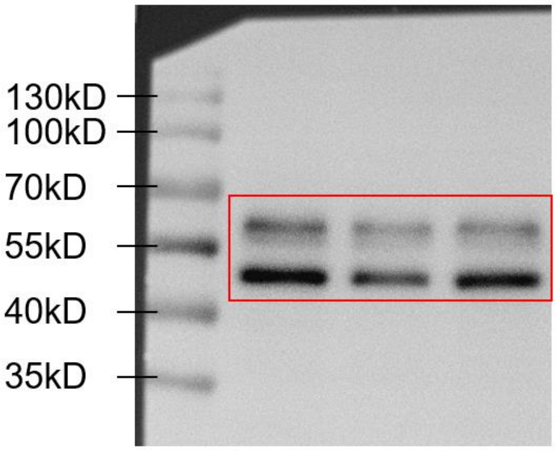


**CaMKII**

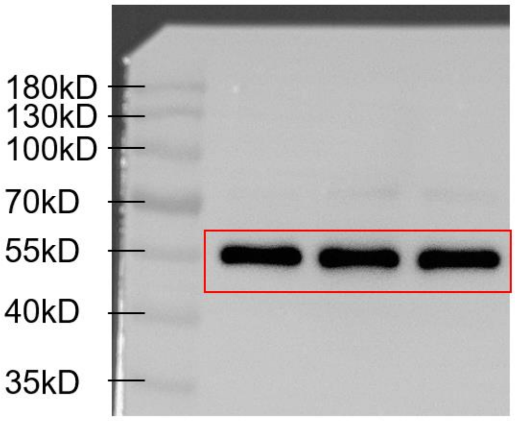


**α-tubulin**

**Figure 5G**

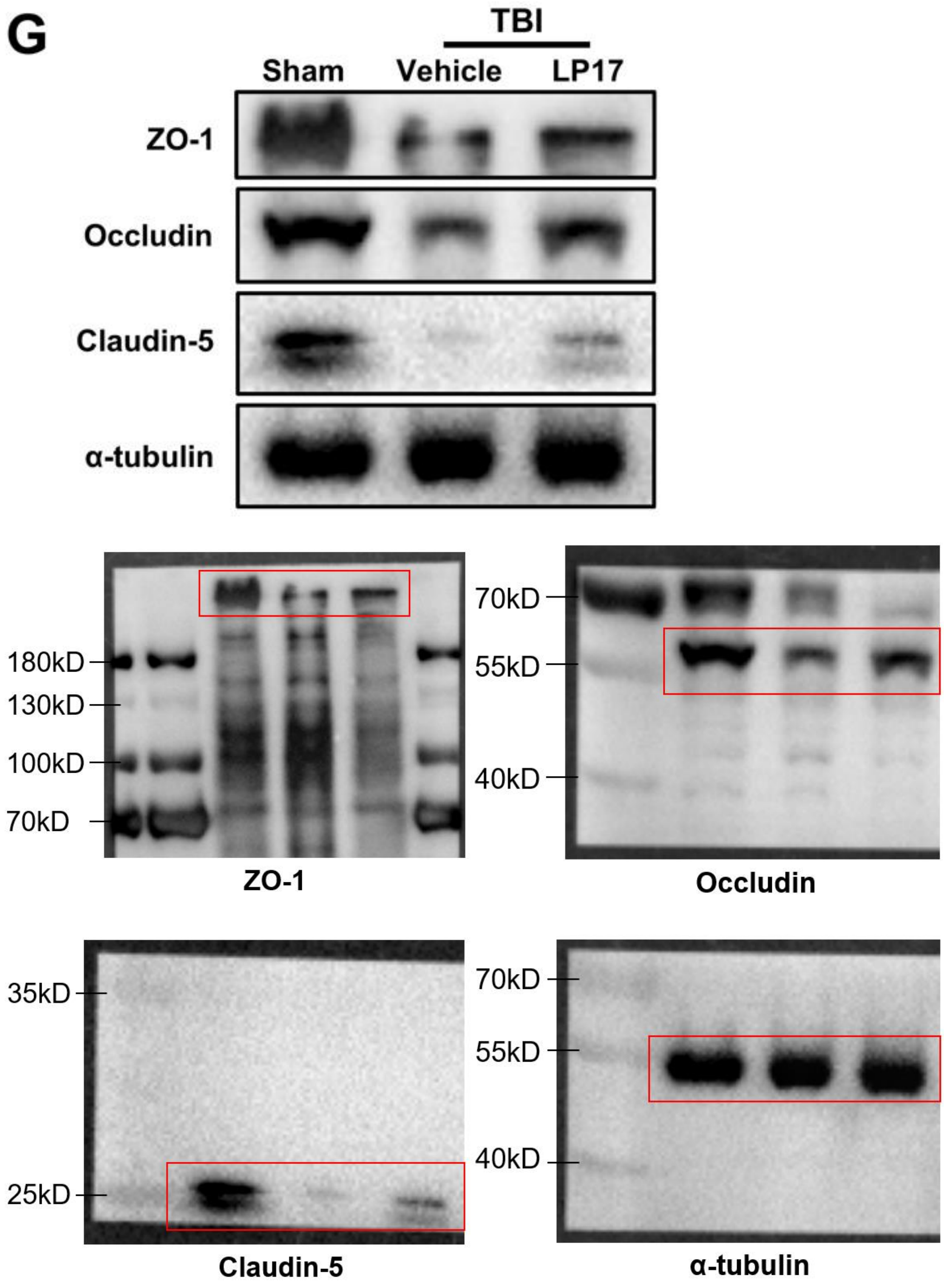
G
TBI
Sham
Vehicle
LP17
ZO-1
Occludin
Claudin-5
α-tubulin
180kD
130kD
100kD
70kD
ZO-1
70kD
55kD
40kD
Occludin
35kD
25kD
Claudin-5
70kD
55kD
40kD
α-tubulin

**Figure 7A**

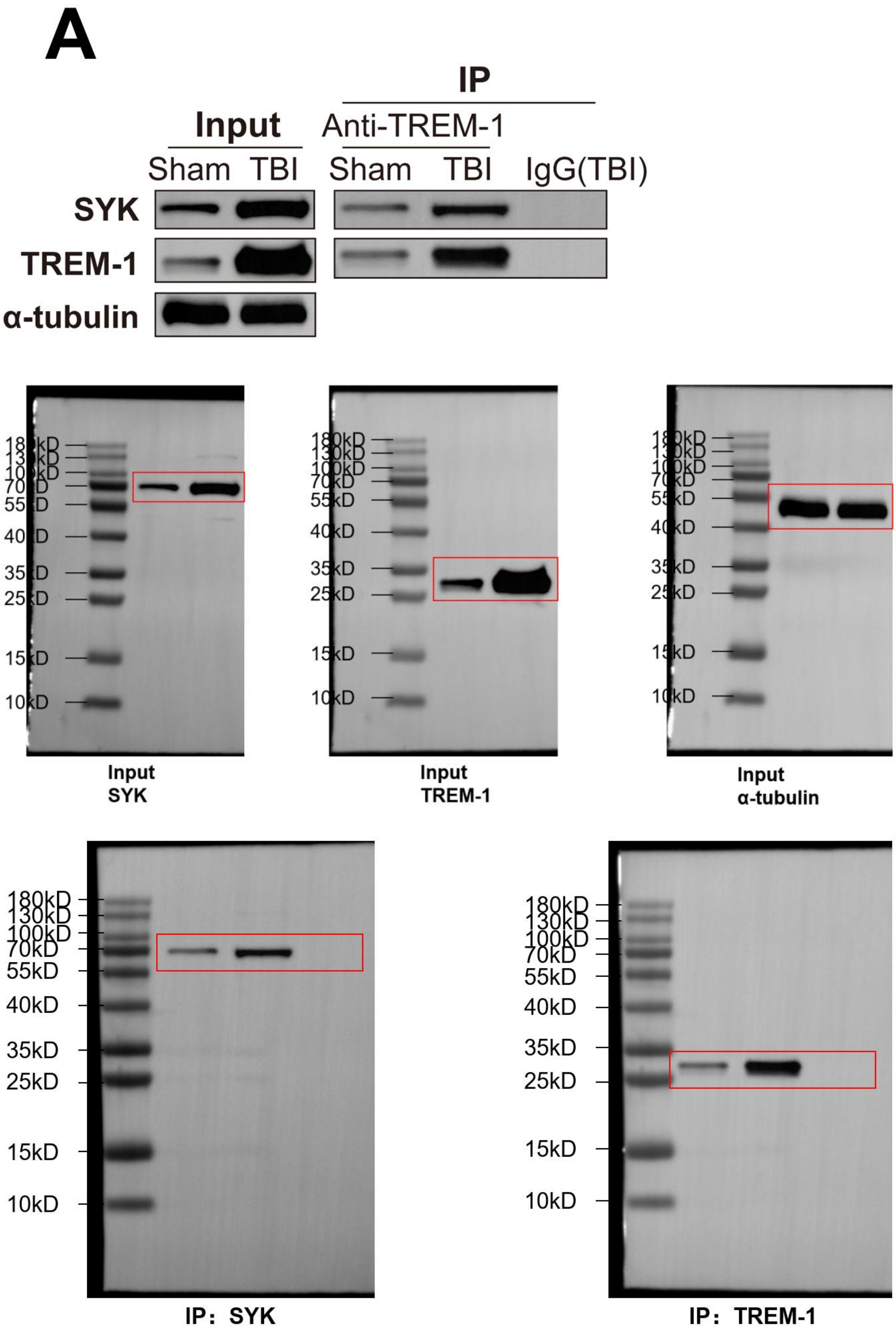
A
IP
Input
Anti-TREM-1
Sham
TBI
Sham
TBI
IgG(TBI)
SYK
TREM-1
α-tubulin
Input
SYK
Input
TREM-1
Input
α-tubulin
180kD
130kD
100kD
70kD
55kD
40kD
35kD
25kD
15kD
10kD
IP：SYK
IP：TREM-1

Figure 7C

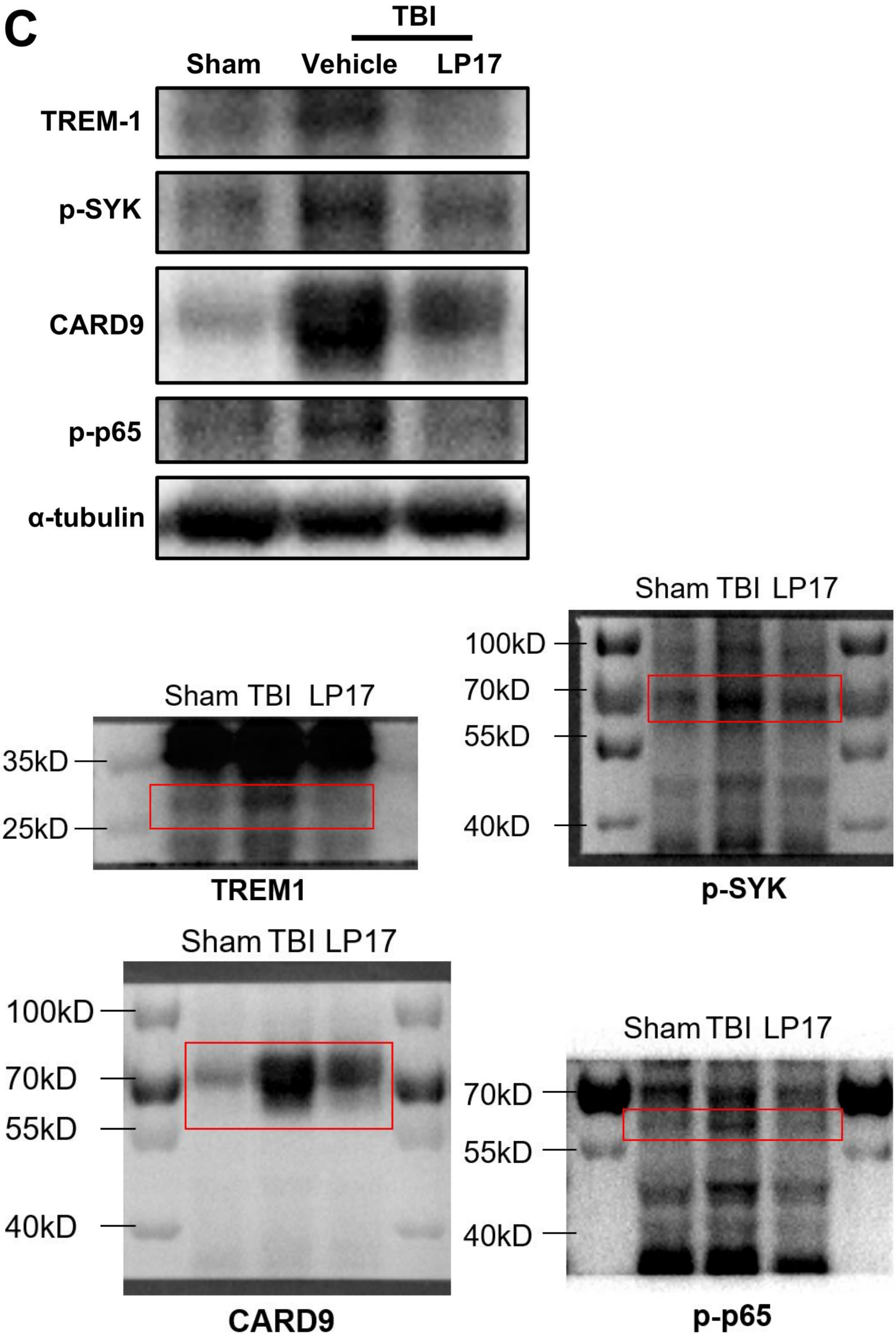
C
TBI
Sham
Vehicle
LP17
TREM-1
p-SYK
CARD9
p-p65
α-tubulin
Sham TBI LP17
35kD
25kD
TREM1
Sham TBI LP17
100kD
70kD
55kD
40kD
p-SYK
Sham TBI LP17
100kD
70kD
55kD
40kD
CARD9
Sham TBI LP17
70kD
55kD
40kD
p-p65

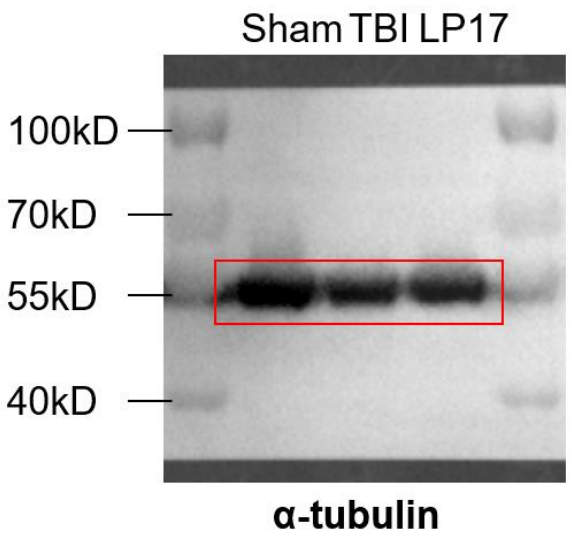
Sham
TBI
LP17
100kD
70kD
55kD
40kD
α-tubulin

## Figure 7D

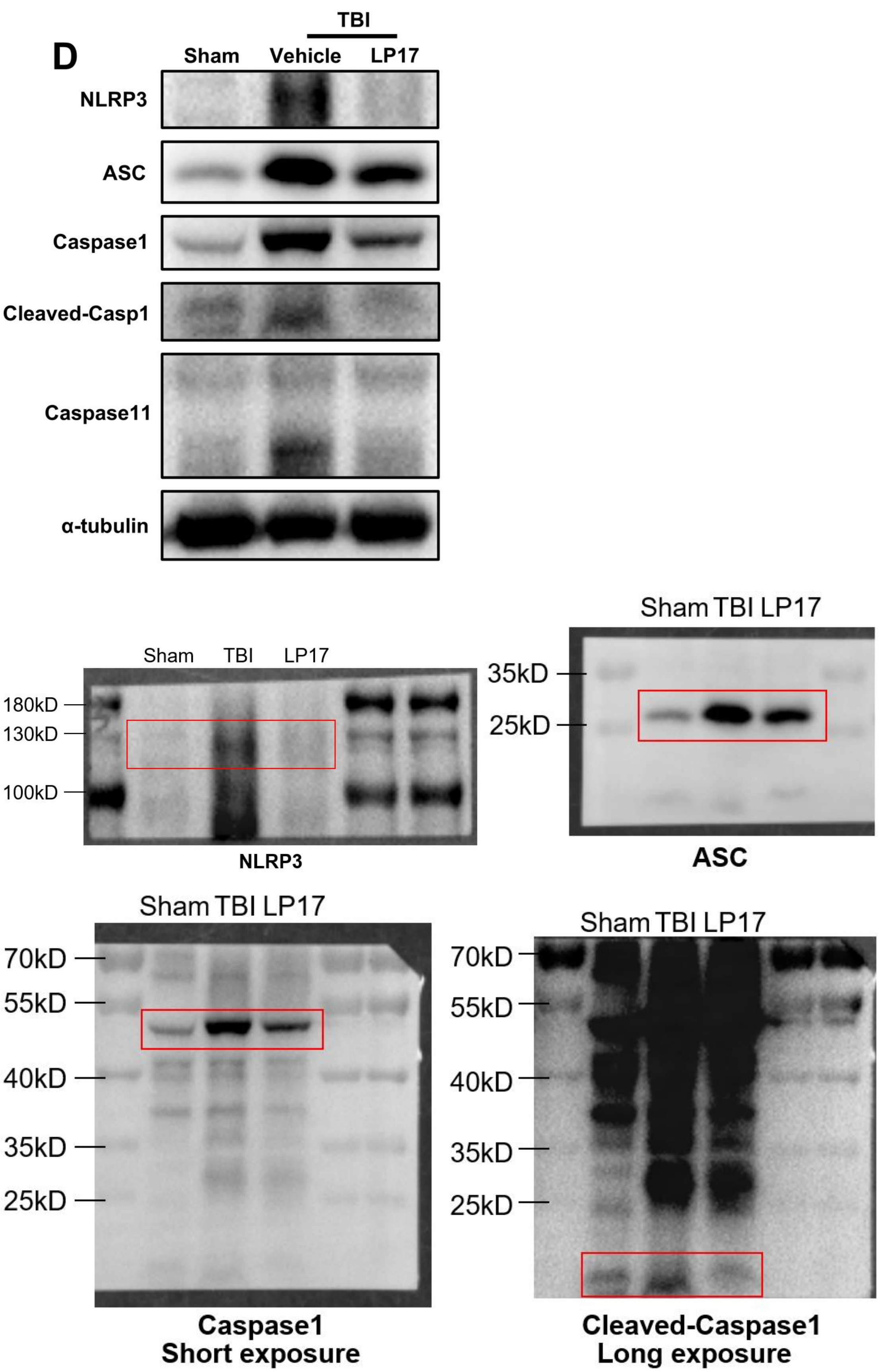
D
TBI
Sham
Vehicle
LP17
NLRP3
ASC
Caspase1
Cleaved-Casp1
Caspase11
α-tubulin
Sham
TBI
LP17
180kD
130kD
100kD
NLRP3
Sham TBI LP17
35kD
25kD
ASC
Sham TBI LP17
70kD
55kD
40kD
35kD
25kD
Caspase1
Short exposure
Sham TBI LP17
70kD
55kD
40kD
35kD
25kD
Cleaved-Caspase1
Long exposure

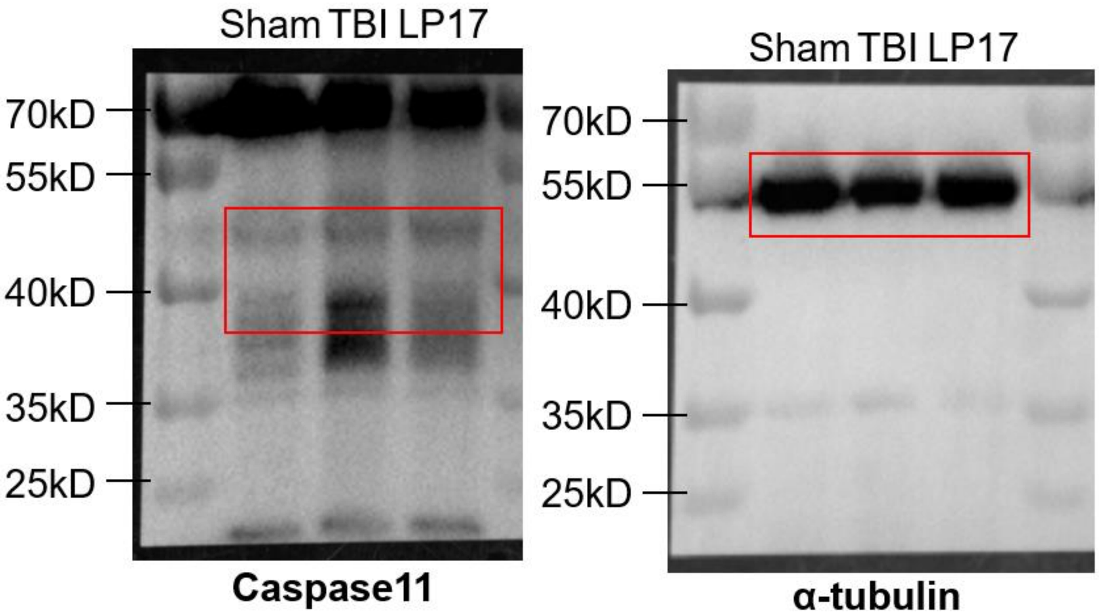
Sham TBI LP17
70kD
55kD
40kD
35kD
25kD
Caspase11
Sham TBI LP17
70kD
55kD
40kD
35kD
25kD
α-tubulin

Figure 8A

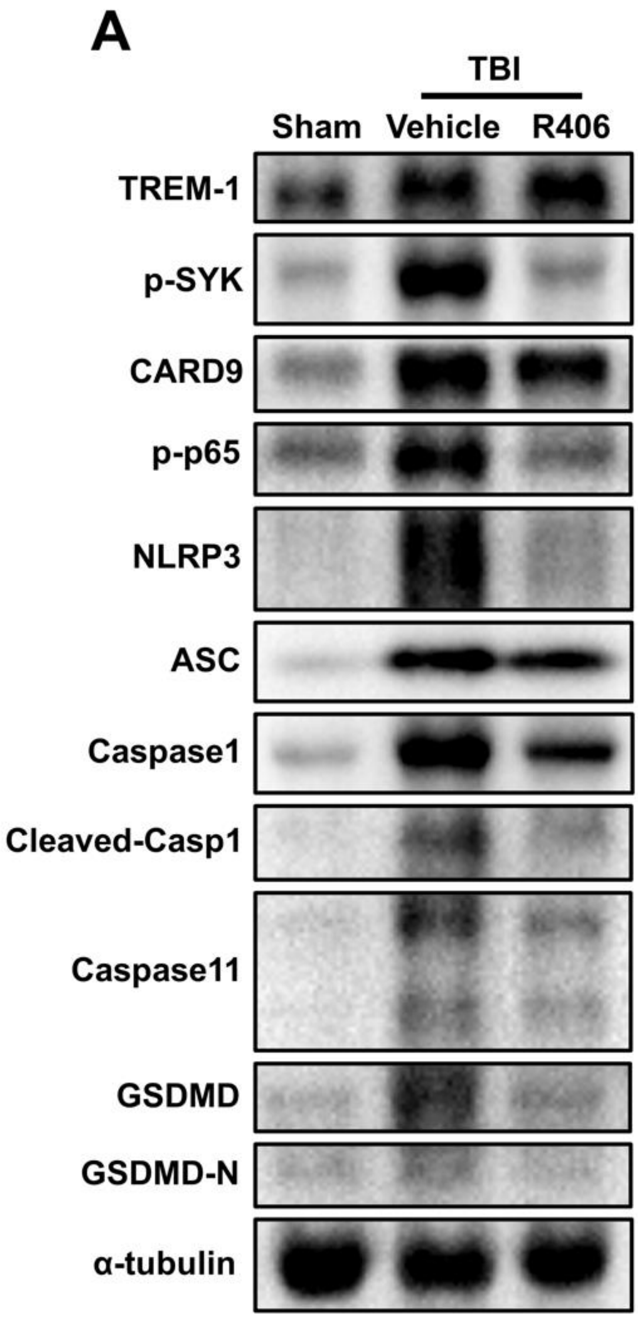
A
TBI
Sham
Vehicle
R406
TREM-1
p-SYK
CARD9
p-p65
NLRP3
ASC
Caspase1
Cleaved-Casp1
Caspase11
GSDMD
GSDMD-N
α-tubulin

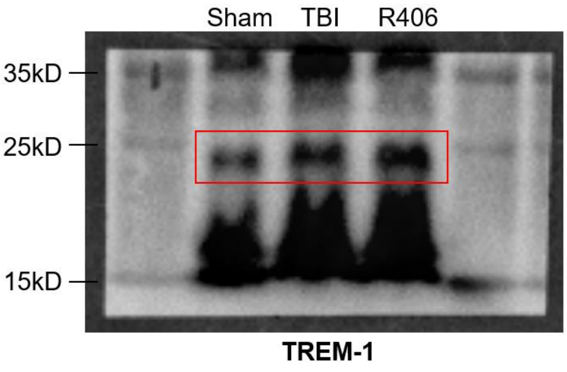
Sham
TBI
R406
35kD
25kD
15kD
TREM-1

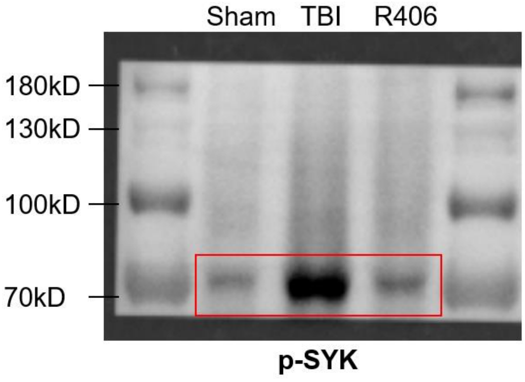
Sham
TBI
R406
180kD
130kD
100kD
70kD
p-SYK

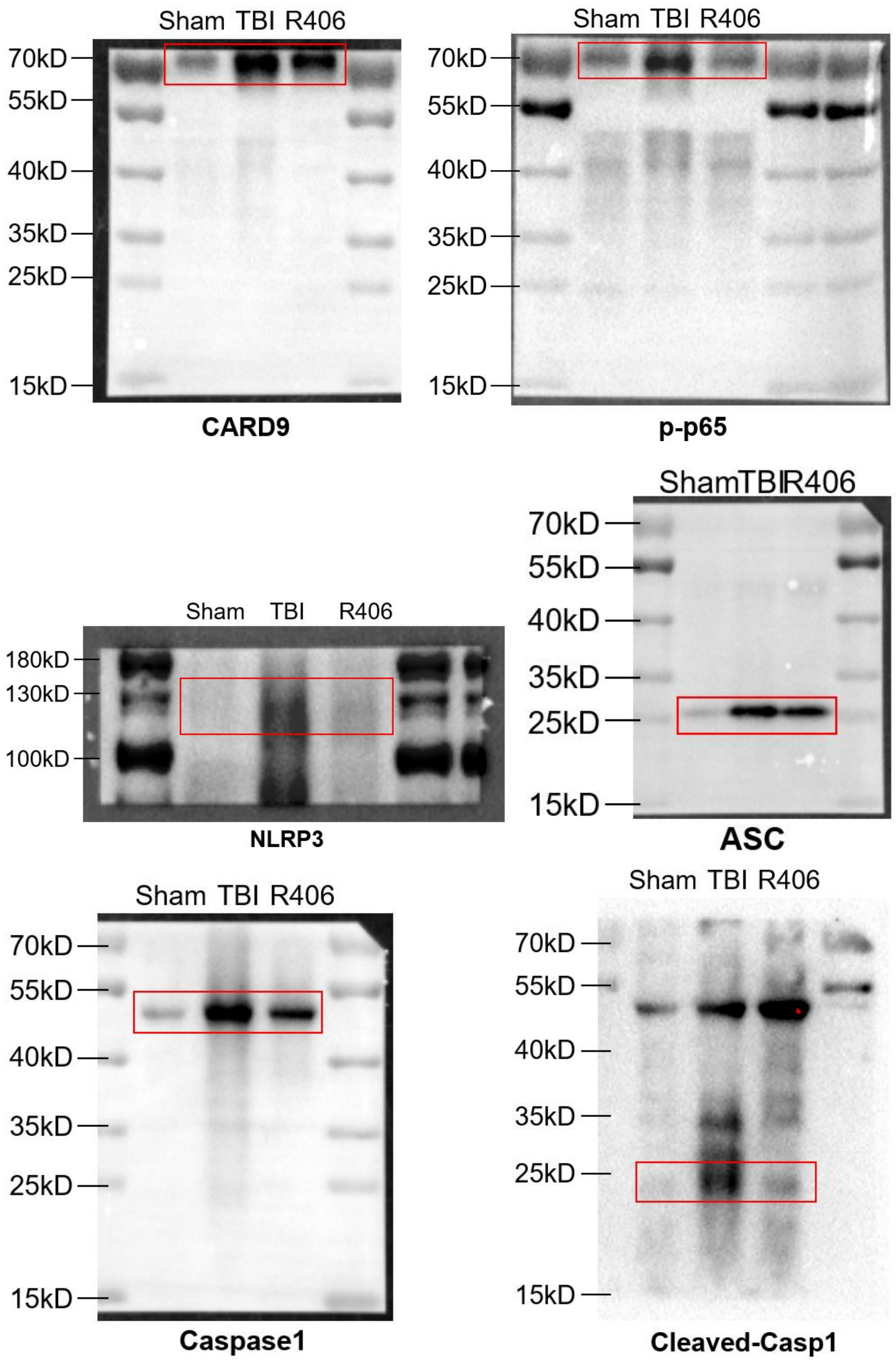

Sham TBI R406
70kD
55kD
40kD
35kD
25kD
15kD
CARD9
Sham TBI R406
70kD
55kD
40kD
35kD
25kD
15kD
p-p65
Sham TBI R406
180kD
130kD
100kD
NLRP3
ShamTBIR406
70kD
55kD
40kD
35kD
25kD
15kD
ASC
Sham TBI R406
70kD
55kD
40kD
35kD
25kD
15kD
Caspase1
Sham TBI R406
70kD
55kD
40kD
35kD
25kD
15kD
Cleaved-Casp1

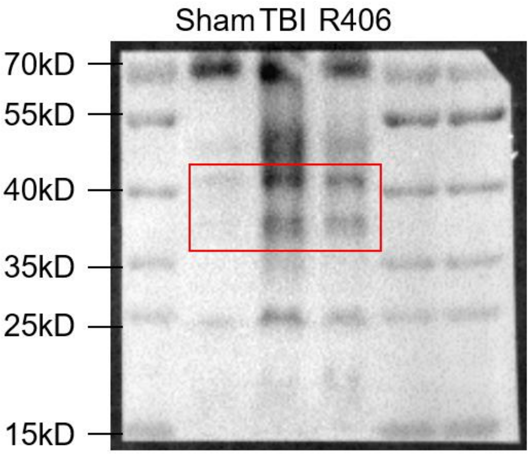


**Caspase11**

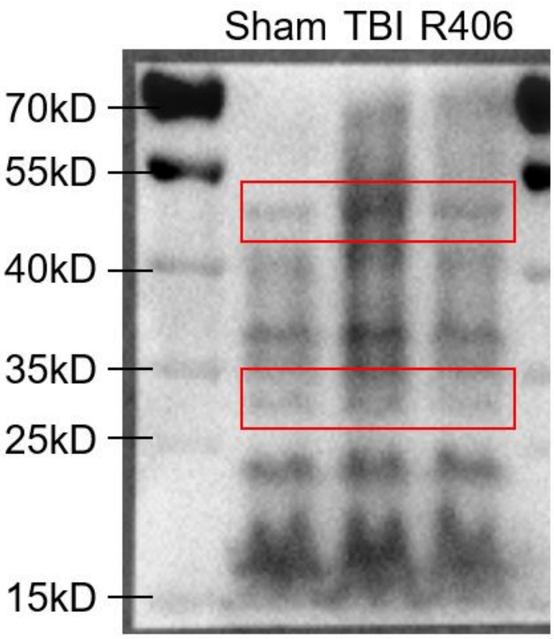


**GSDMD and GSDMD-N**

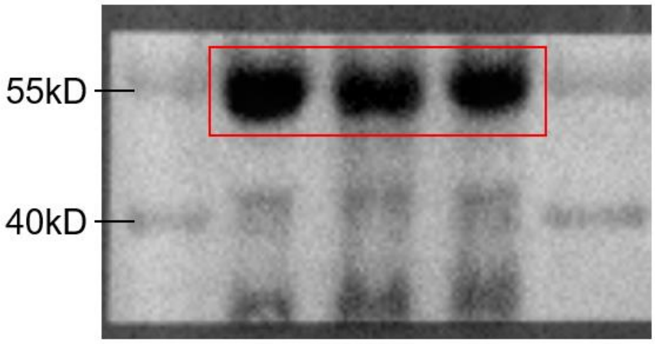


**α-tubulin**

Figure 9A

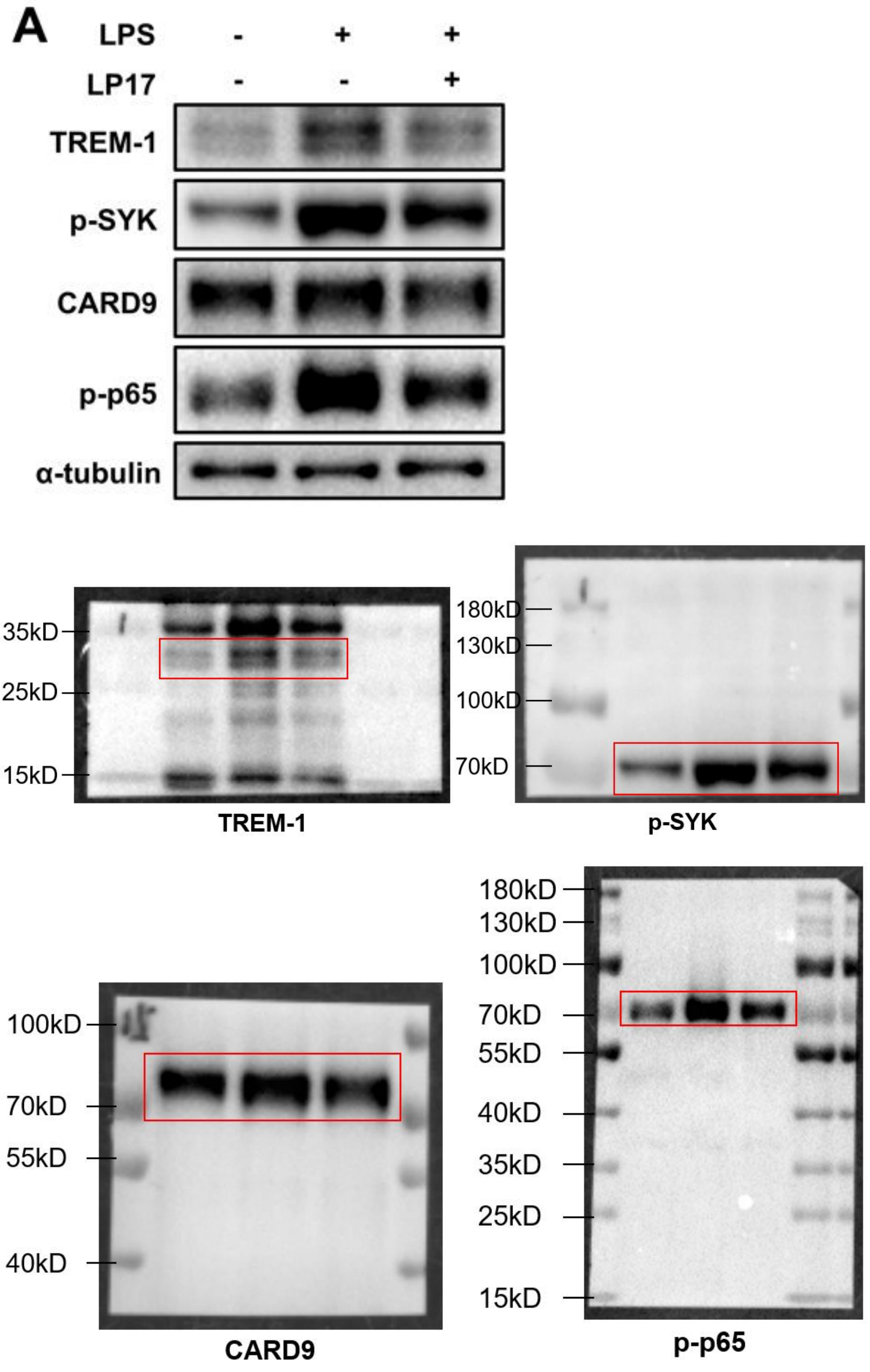

A
LPS
-
+
+
LP17
-
-
+
TREM-1
p-SYK
CARD9
p-p65
α-tubulin
35kD
25kD
15kD
TREM-1
180kD
130kD
100kD
70kD
p-SYK
100kD
70kD
55kD
40kD
CARD9
180kD
130kD
100kD
70kD
55kD
40kD
35kD
25kD
15kD
p-p65

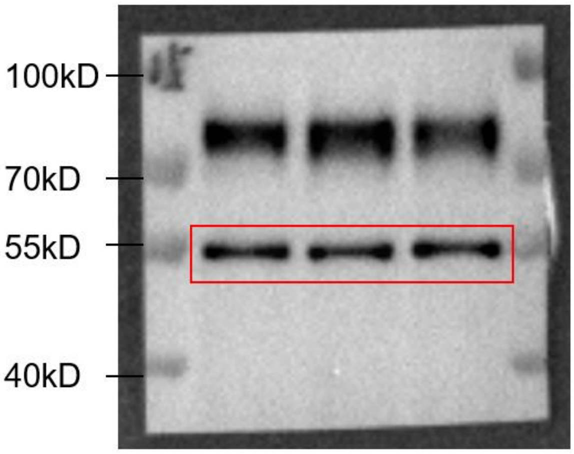

**α-tubulin**

**Figure 9F**

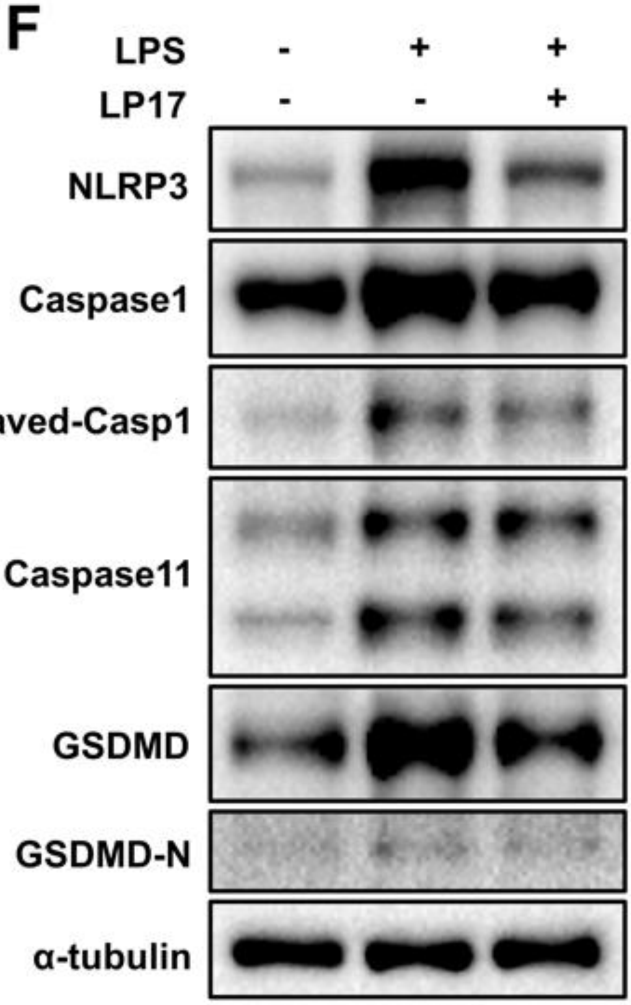


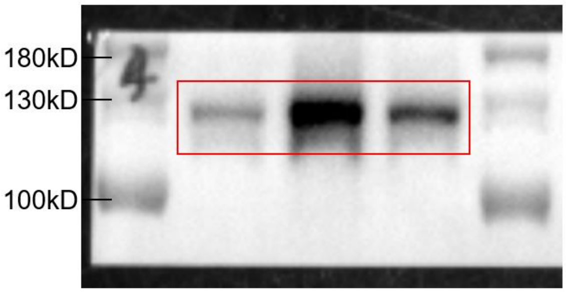

NLRP3

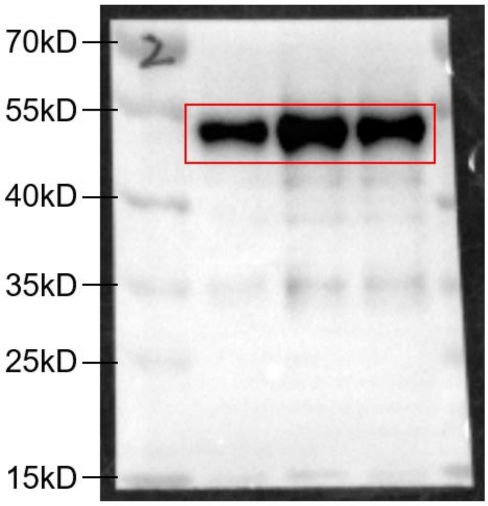

Caspase1

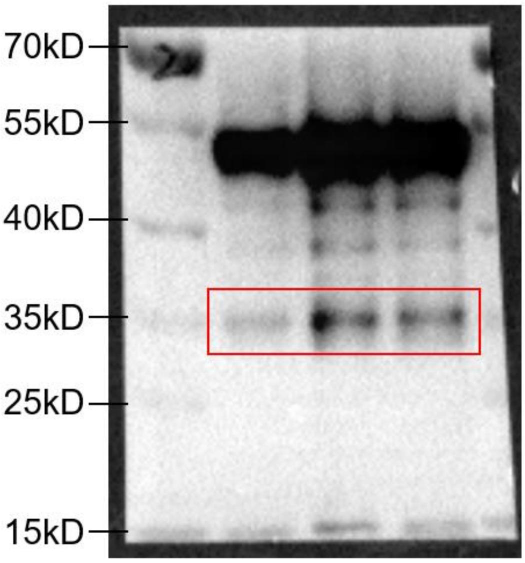


**Cleaved-Casp1**

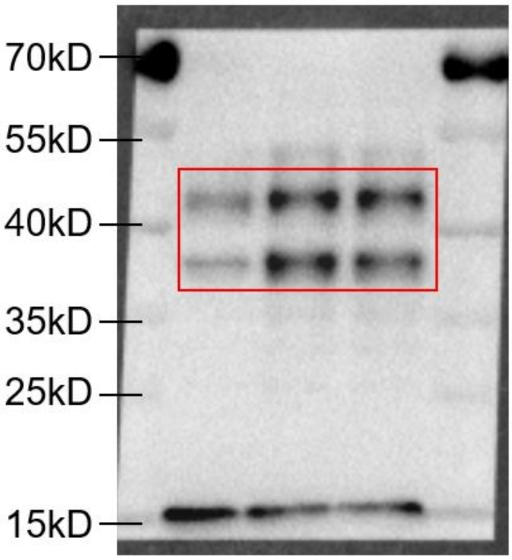


**Caspase11**

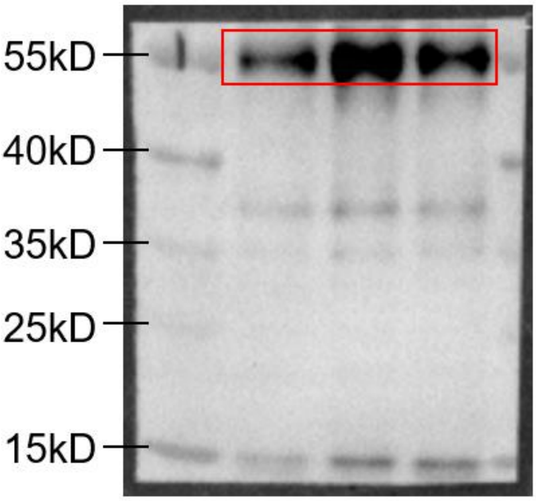


**GSDMD**

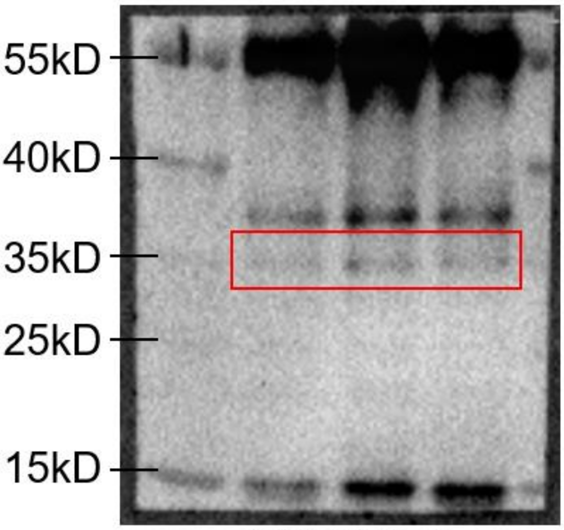


**GSDMD-N**

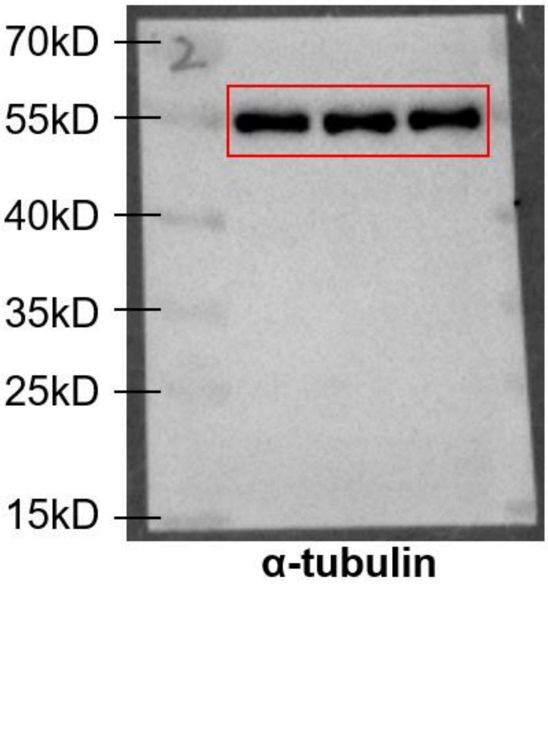


**α-tubulin**

Figure 10A

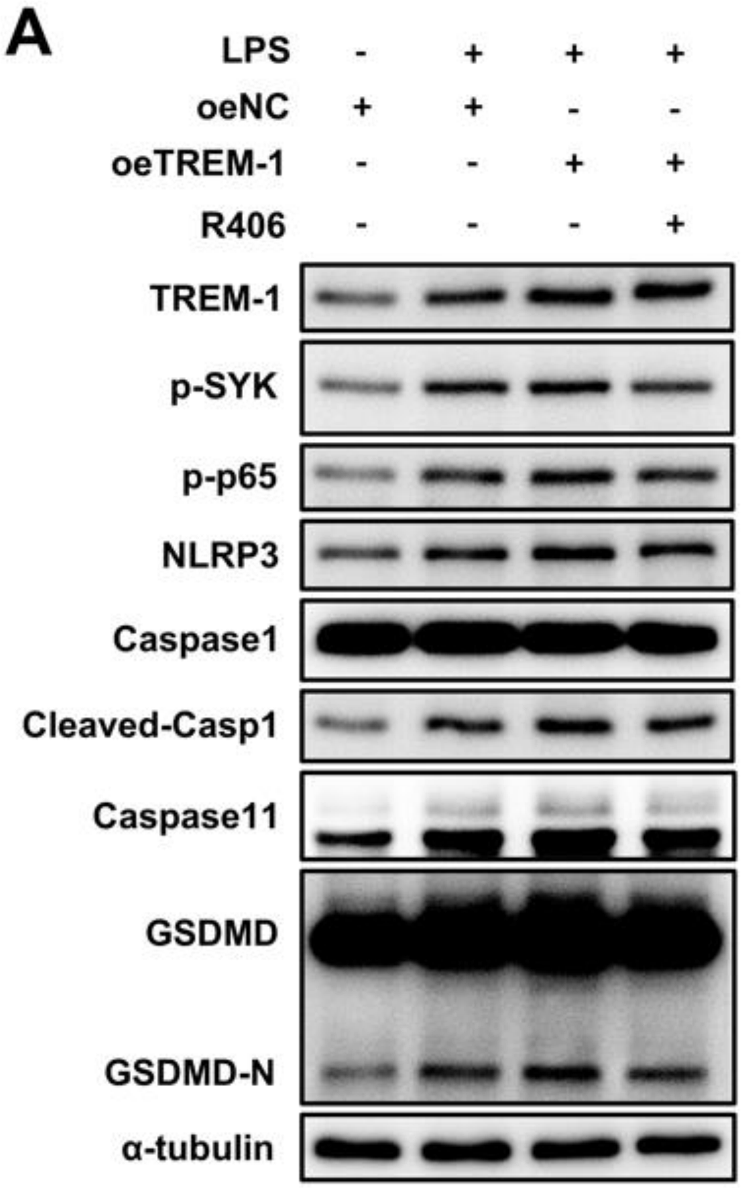


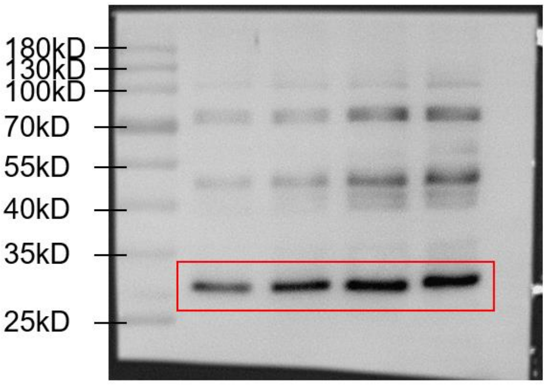


TREM-1

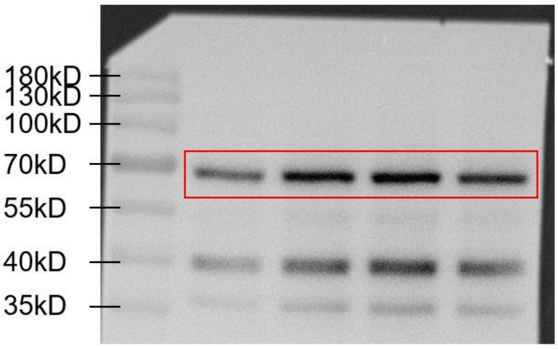


p-SYK

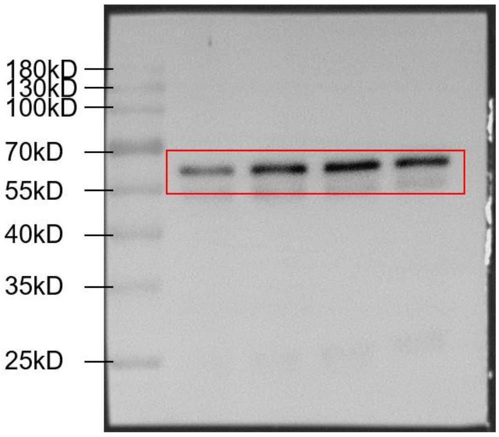


**p-p65**

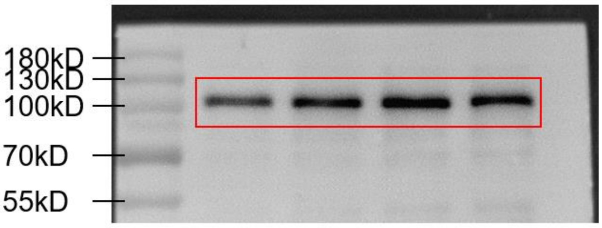


**NLRP3**

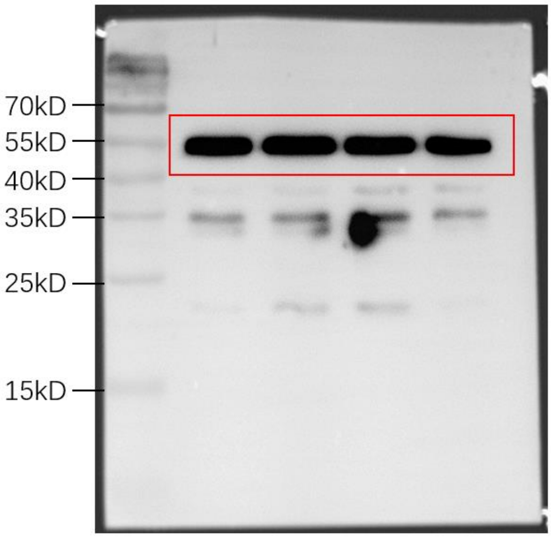


**Caspase1**

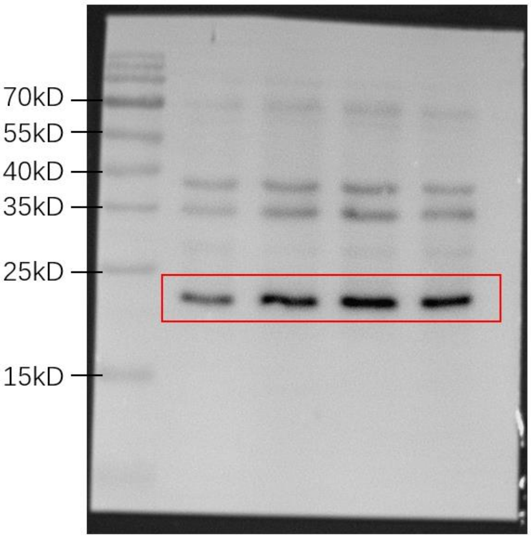


**Cleaved-Casp1**

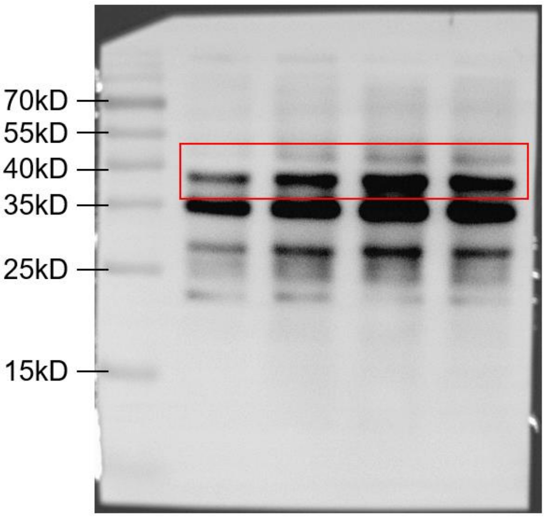


**Caspase11**

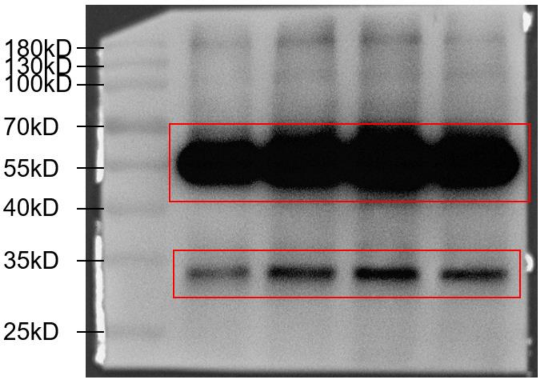


**GSDMD and GSDMD-N**

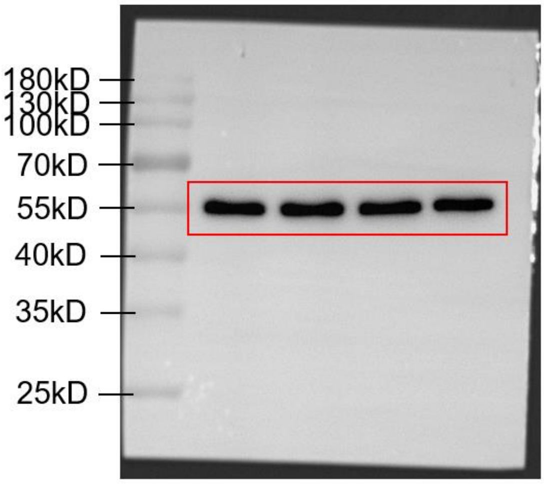

**α-tubulin**

**Supplementary Figure 4A**

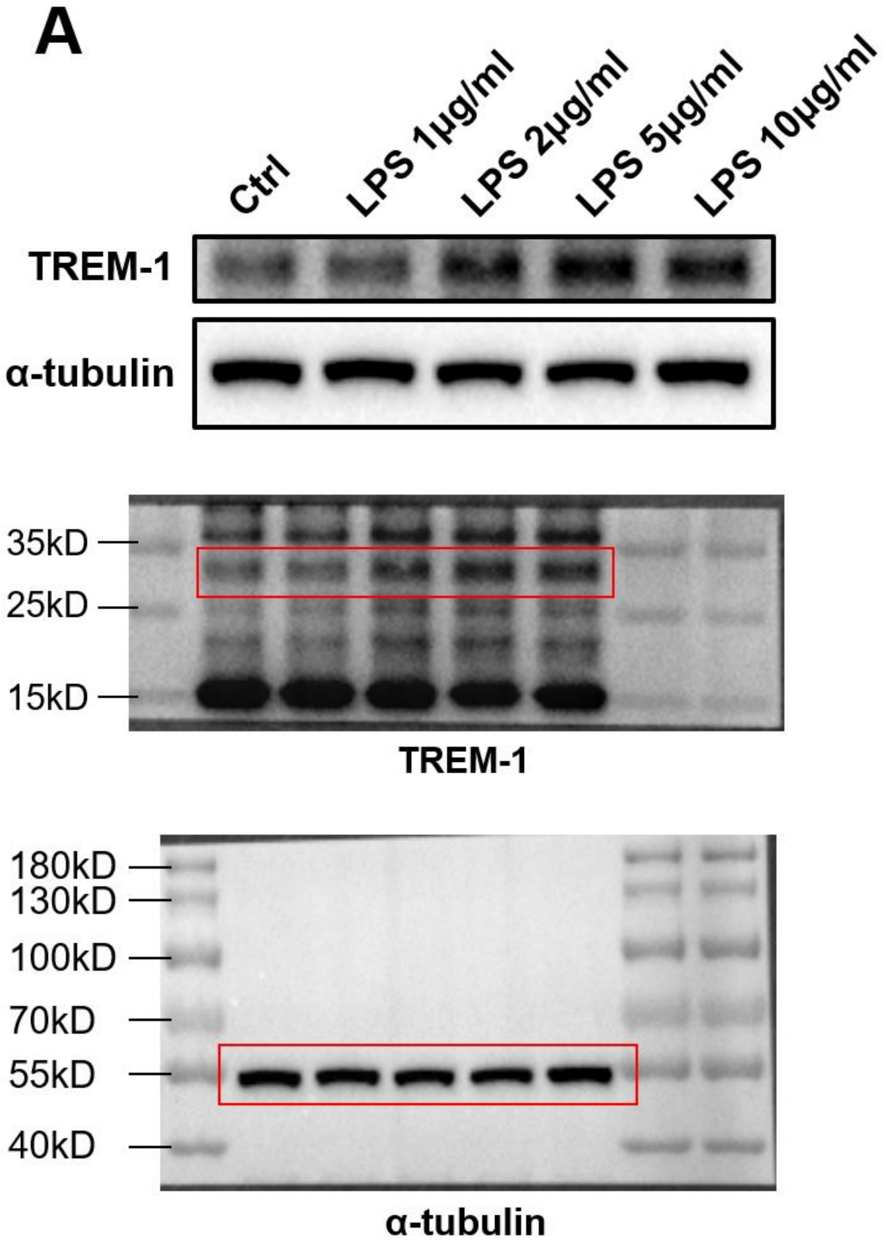

**Supplementary Figure 4B**

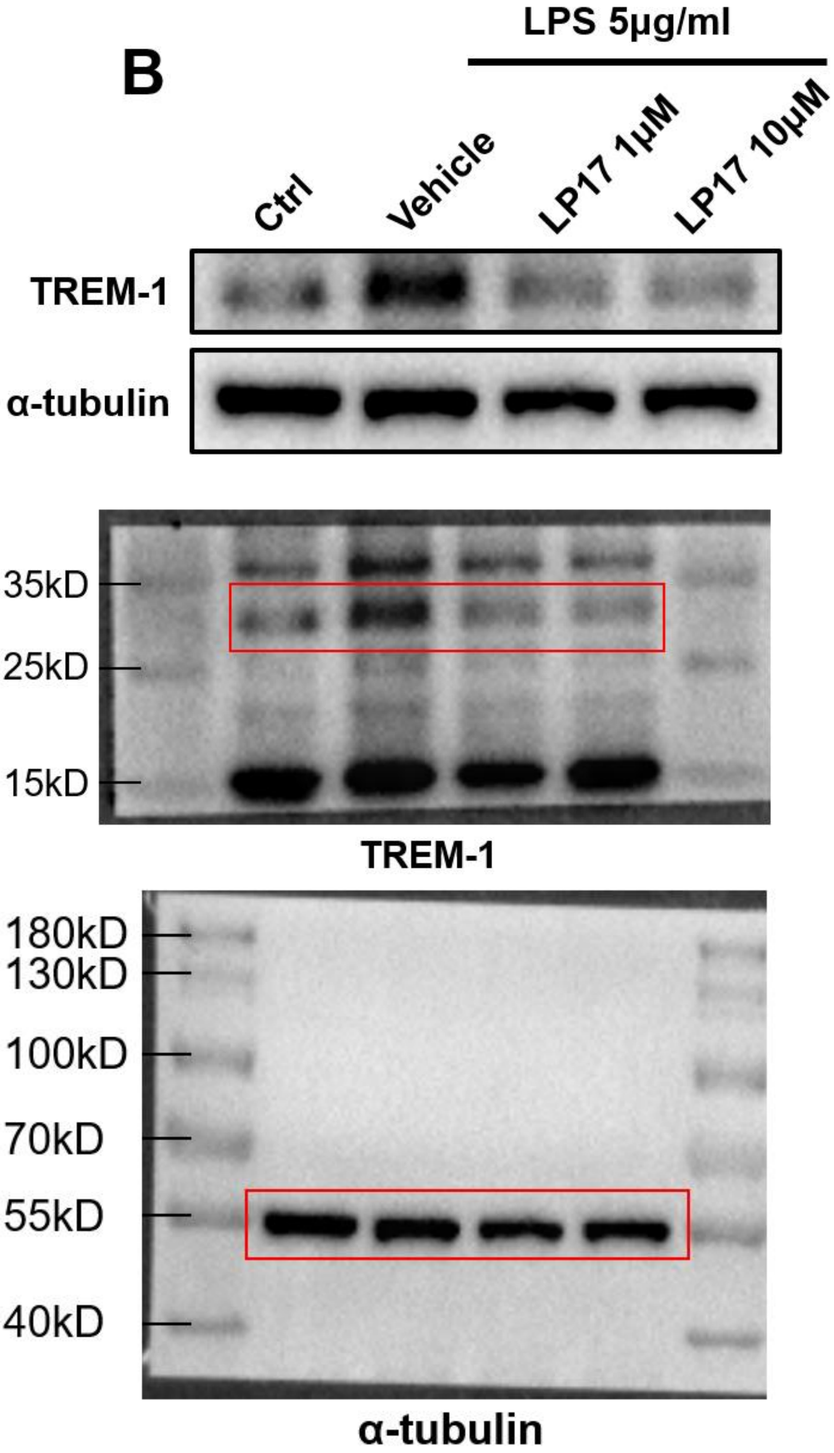

**Supplementary Figure 5A**

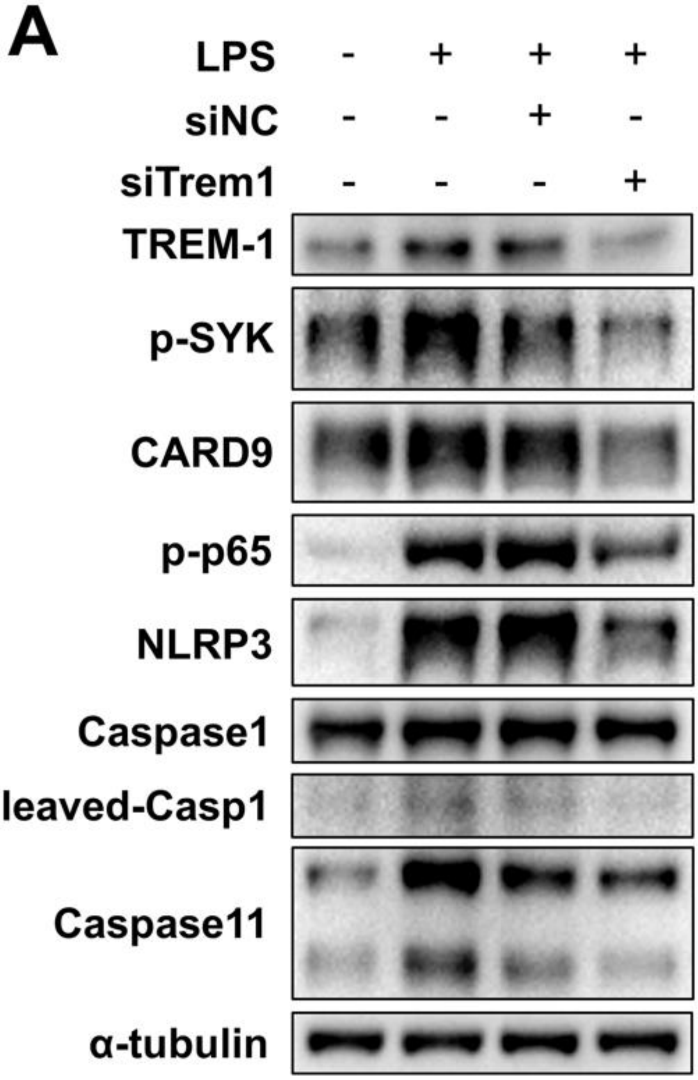


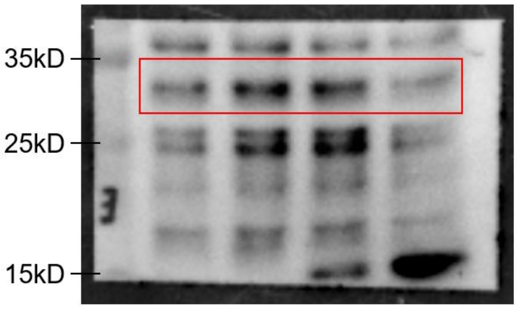


TREM-1

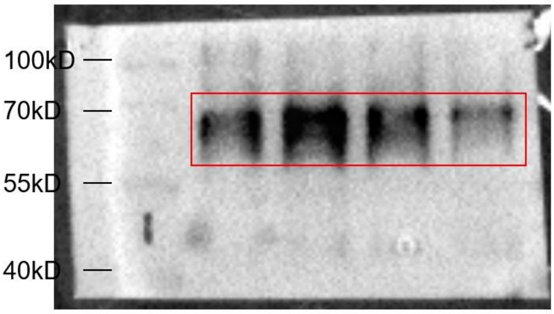


p-SYK

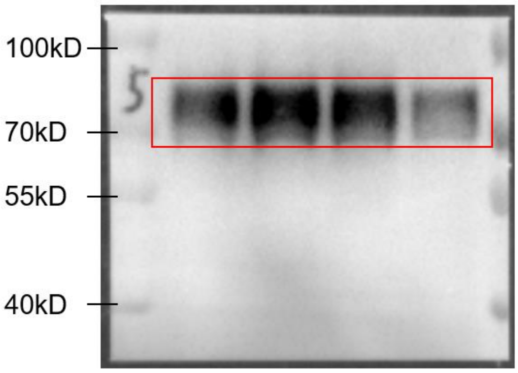

**CARD9**

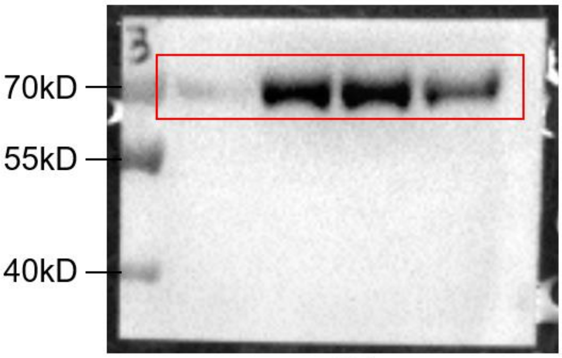

**p-p65**

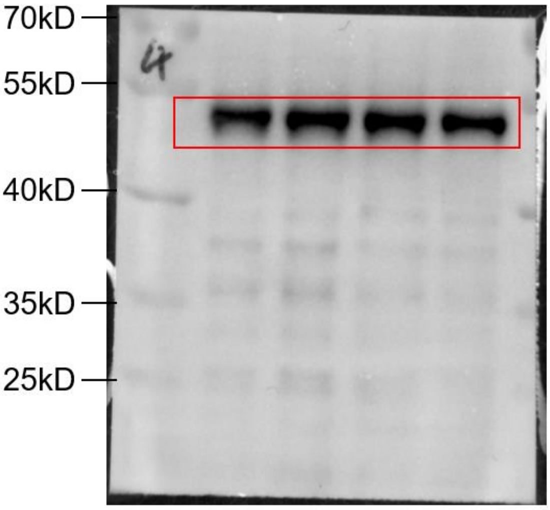

**Caspase1**

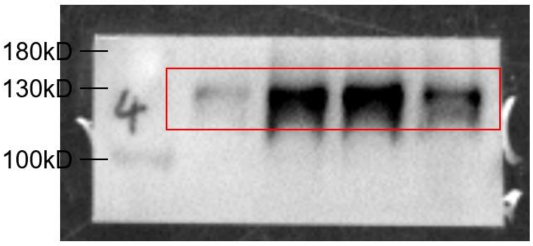

**NLRP3**

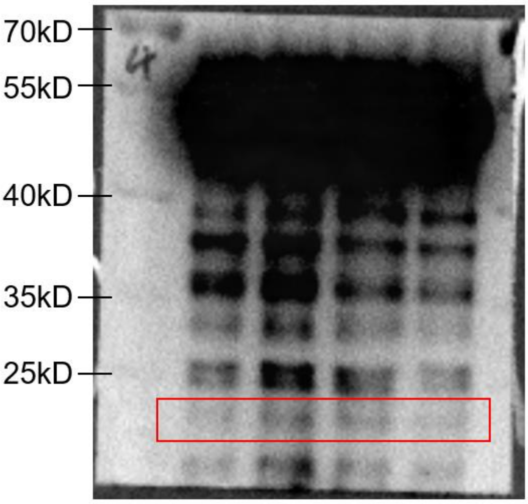

**Cleaved-Casp1**

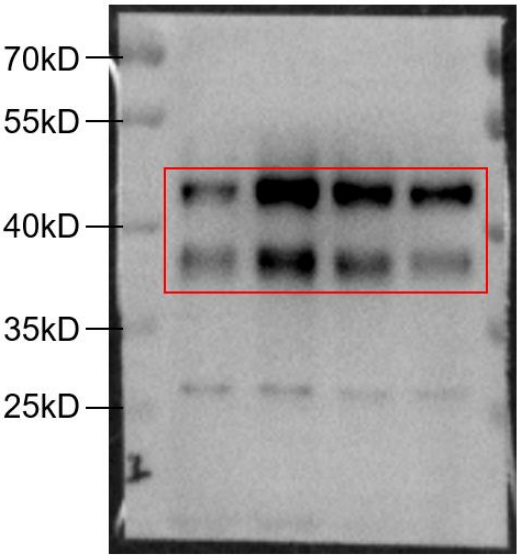

**Caspase11**

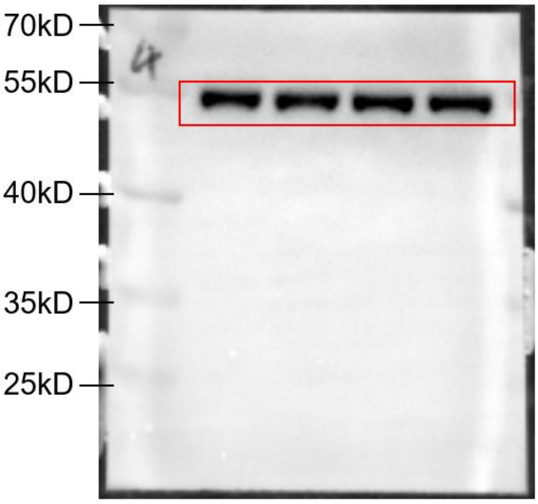


**α-tubulin**
