# Supplementary figures and images for: TREM-1 as a potential gatekeeper of neuroinflammatory responses: therapeutic validation and mechanistic insights in experimental traumatic brain injury
Source: Front Immunol. 2025 Jul 21;16:1636917. doi: 10.3389/fimmu.2025.1636917 (PMC12318749; doi:10.3389/fimmu.2025.1636917)

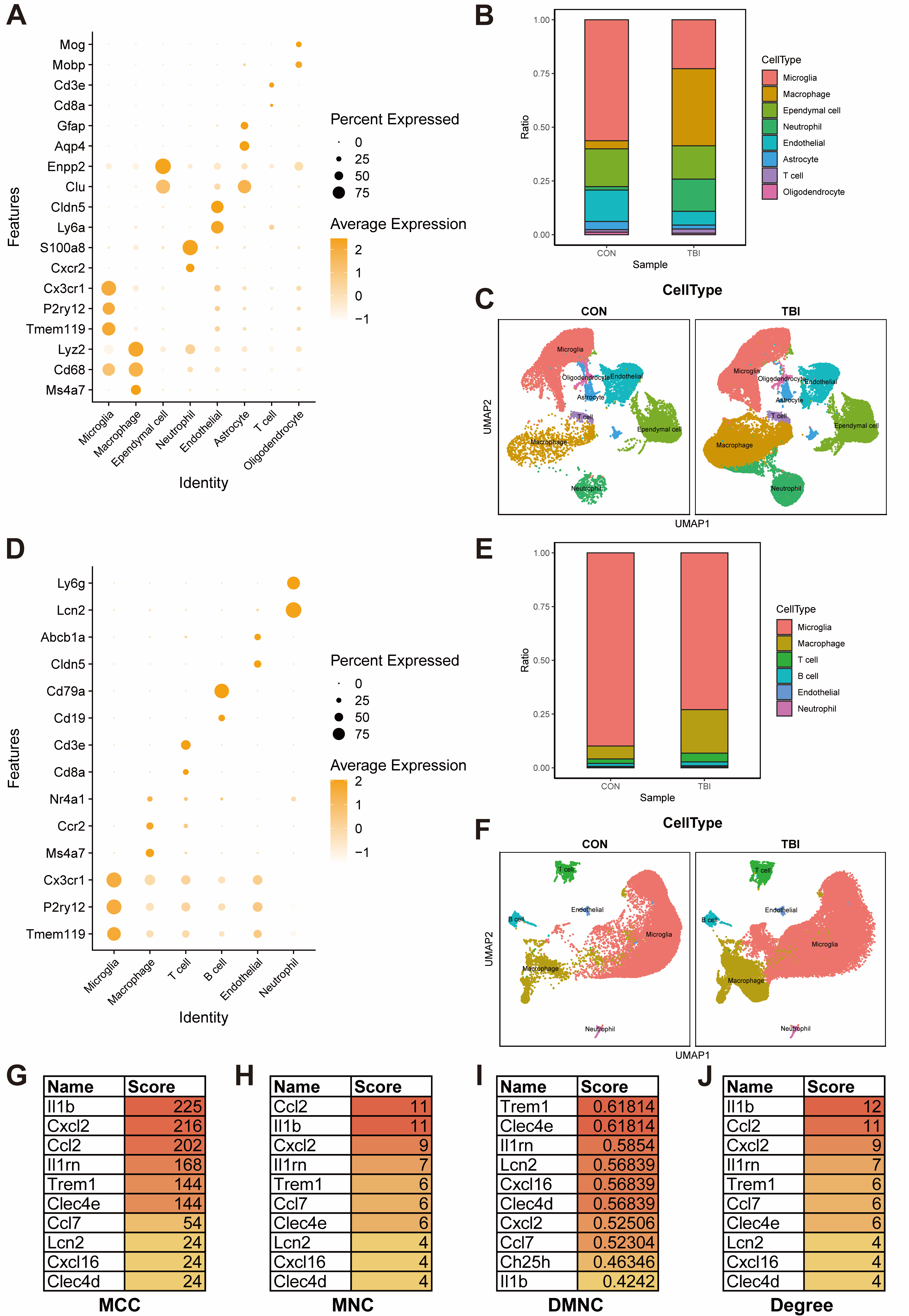

Supplement: Supplementary file 1 [file DataSheet1.zip › Supplementary Material/Supplementary Figure 2.tif]

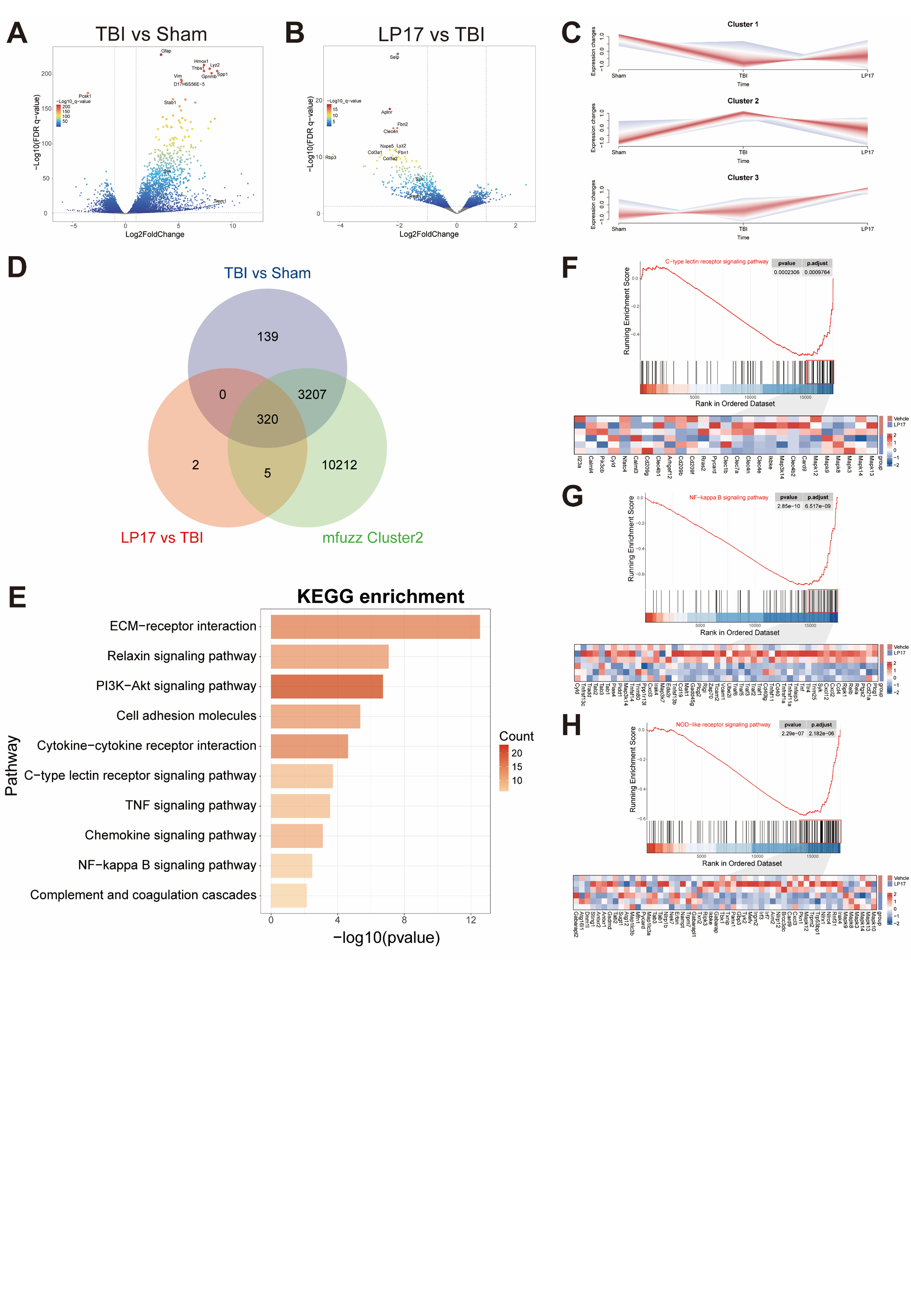

Supplement: Supplementary file 1 [file DataSheet1.zip › Supplementary Material/Supplementary Figure 3.tif]

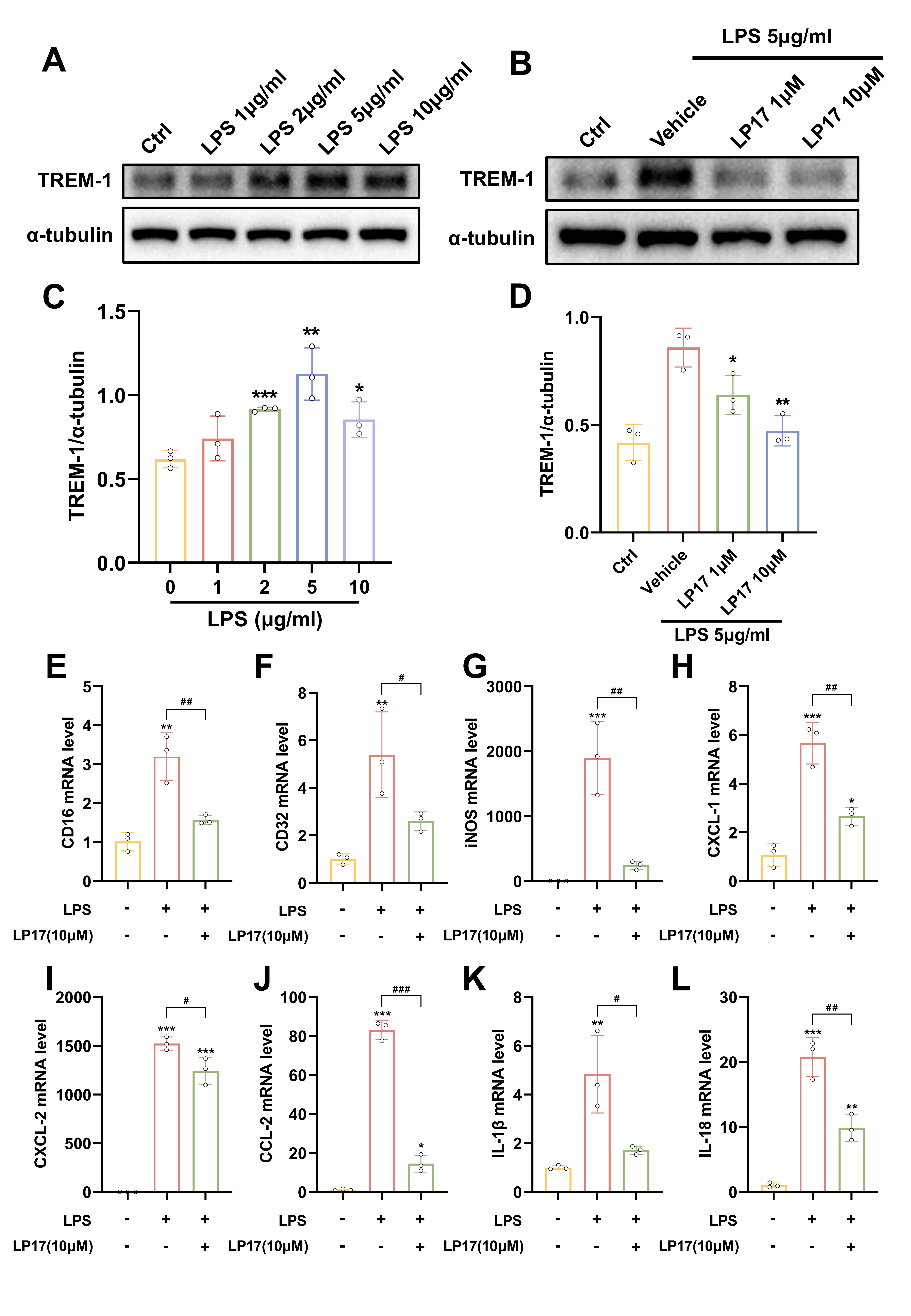

Supplement: Supplementary file 1 [file DataSheet1.zip › Supplementary Material/Supplementary Figure 4.tif]

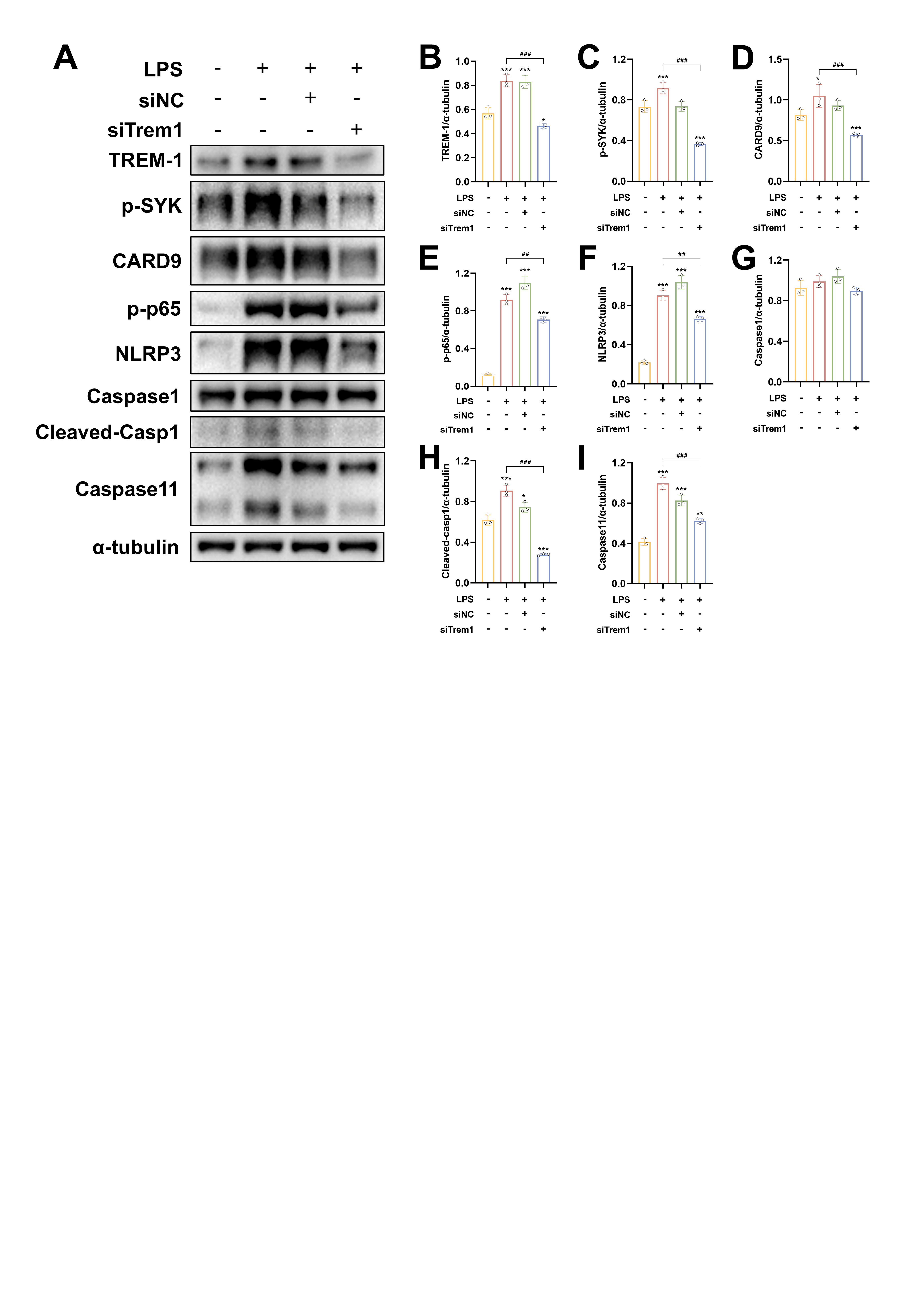

Supplement: Supplementary file 1 [file DataSheet1.zip › Supplementary Material/Supplementary Figure 5.tif]
